# Trends in outpatient and inpatient visits for separate ambulatory-care-sensitive conditions during the first year of the COVID-19 pandemic: a province-based study

**DOI:** 10.3389/fpubh.2023.1251020

**Published:** 2023-12-18

**Authors:** Tetyana Kendzerska, David T. Zhu, Michael Pugliese, Douglas Manuel, Mohsen Sadatsafavi, Marcus Povitz, Therese A. Stukel, Teresa To, Shawn D. Aaron, Sunita Mulpuru, Melanie Chin, Claire E. Kendall, Kednapa Thavorn, Rebecca Robillard, Andrea S. Gershon

**Affiliations:** ^1^The Ottawa Hospital Research Institute, Ottawa, ON, Canada; ^2^Department of Medicine, Faculty of Medicine, University of Ottawa, ON, Canada; ^3^ICES, Ottawa, Toronto, ON, Canada; ^4^Medical Scientist Training Program, School of Medicine, Virginia Commonwealth University, Richmond, VA, United States; ^5^Respiratory Evaluation Sciences Program, Faculty of Pharmaceutical Sciences, The University of British Columbia, Vancouver, BC, Canada; ^6^Department of Medicine, Cumming School of Medicine, University of Calgary, Calgary, AB, Canada; ^7^Sunnybrook Research Institute, Sunnybrook Health Sciences Centre, Toronto, ON, Canada; ^8^Institute of Health Policy, Management and Evaluation, University of Toronto, Toronto, ON, Canada; ^9^Dalla Lana School of Public Health, University of Toronto, Toronto, ON, Canada; ^10^Research Institute, The Hospital of Sick Children, Toronto, ON, Canada; ^11^Bruyère Research Institute, Ottawa, ON, Canada; ^12^The Department of Family Medicine, University of Ottawa, Ottawa, ON, Canada; ^13^Faculty of Medicine, School of Epidemiology and Public Health, University of Ottawa, Ottawa, ON, Canada; ^14^School of Psychology, University of Ottawa, Ottawa, ON, Canada; ^15^Department of Medicine, University of Toronto, Toronto, ON, Canada

**Keywords:** COVID-19 pandemic, ambulatory care sensitive conditions, outpatient visits, inpatient visits, ARIMA, trends

## Abstract

**Background:**

The COVID-19 pandemic led to global disruptions in non-urgent health services, affecting health outcomes of individuals with ambulatory-care-sensitive conditions (ACSCs).

**Methods:**

We conducted a province-based study using Ontario health administrative data (Canada) to determine trends in outpatient visits and hospitalization rates (per 100,000 people) in the general adult population for seven ACSCs during the first pandemic year (March 2020–March 2021) compared to previous years (2016–2019), and how disruption in outpatient visits related to acute care use. ACSCs considered were chronic obstructive pulmonary disease (COPD), asthma, angina, congestive heart failure (CHF), hypertension, diabetes, and epilepsy. We used time series auto-regressive integrated moving-average models to compare observed versus projected rates.

**Results:**

Following an initial reduction (March–May 2020) in all types of visits, primary care outpatient visits (combined in-person and virtual) returned to pre-pandemic levels for asthma, angina, hypertension, and diabetes, remained below pre-pandemic levels for COPD, and rose above pre-pandemic levels for CHF (104.8 vs. 96.4, 95% CI: 89.4–104.0) and epilepsy (29.6 vs. 24.7, 95% CI: 22.1–27.5) by the end of the first pandemic year. Specialty visits returned to pre-pandemic levels for COPD, angina, CHF, hypertension, and diabetes, but remained above pre-pandemic levels for asthma (95.4 vs. 79.5, 95% CI: 70.7–89.5) and epilepsy (53.3 vs. 45.6, 95% CI: 41.2–50.5), by the end of the year. Virtual visit rates increased for all ACSCs. Among ACSCs, reductions in hospitalizations were most pronounced for COPD and asthma. CHF-related hospitalizations also decreased, albeit to a lesser extent. For angina, hypertension, diabetes, and epilepsy, hospitalization rates reduced initially, but returned to pre-pandemic levels by the end of the year.

**Conclusion:**

This study demonstrated variation in outpatient visit trends for different ACSCs in the first pandemic year. No outpatient visit trends resulted in increased hospitalizations for any ACSC; however, reductions in rates of asthma, COPD, and CHF hospitalizations persisted.

## 1 Introduction

The COVID-19 pandemic led to significant disruptions in the accessibility and provision of non-urgent health services in Canada and globally that affected the management and health outcomes of patients (1). Ambulatory-care-sensitive conditions (ACSCs) are health conditions for which timely access to outpatient care and effective management is known to improve prognosis and prevent emergency department (ED) visits and hospitalizations (2). Thus, inpatient visits for ACSCs are usually accepted as an indicator of the quality and accessibility of outpatient care (3).

Previous studies on the impact of the COVID-19 pandemic on the management, treatment, and care of ACSC patients were limited in several ways: they focused only on the initial parts of the pandemic (4, 5) and failed to note trends once events had settled down and became more routine; they typically focused on a specific type of health service use (i.e., ED visits or hospitalizations) without exploring how disruption in outpatient visits related to acute care use, and either examined an individual single chronic disease without comparing trends between different chronic conditions or aggregated “chronic diseases” as an outcome (6–13).

In our recent population-based study, we did not observe an increase in ED visits or hospital admission rates for all ACSCs combined in a general adult population during the first year of the pandemic, despite a reduction in outpatient visit rates at the beginning of the pandemic (12). These findings were corroborated by global trends in chronic disease health service utilization during the COVID-19 pandemic (14–18). However, we may have failed to find trends for individual diseases in our aggregate results (12) due to disease-specific healthcare needs, natural history, development, and progression (19).

Therefore, to address gaps in the literature and understand how disruption in outpatient visits related to acute care use, we conducted a province-based cohort study to compare actual (observed) and projected (counterfactual estimates based on pre-COVID-19 pandemic periods) trends in outpatient and inpatient visits for seven separate ACSCs – asthma, chronic obstructive pulmonary disease (COPD), angina, congestive heart failure (CHF), hypertension, diabetes mellitus, and epilepsy – in the general adult population. We hypothesized a steep reduction in outpatient and inpatient visits for seven separate ACSCs during the initial phase of the pandemic (March–May 2020) compared to the pre-pandemic data, followed by a slow recovery during the later phase of the pandemic. We also hypothesize variations in trends for different ACSCs.

## 2 Materials and methods

### 2.1 Study design

We used provincial health administrative data from Ontario, Canada, during the first year of the COVID-19 pandemic (March 2020–March 2021) to compare to similar periods in previous years (January 2016–December 2019). Ontario is the most populous province of Canada, with a population of more than 14.5 million (20) and universal health insurance.

ICES is an independent non-profit institution and a prescribed entity under Personal Health Information Protection Act (PHIPA) in Ontario. Under section 45 of PHIPA, ICES is authorized to collect personal health information without consent to compile statistical information or analysis concerning managing, allocating resources to, monitoring, evaluating or planning for the health system. Projects that use health administrative databases collected by ICES under section 45 of PHIPA are exempt from REB review. This study was authorized under section 45 of PHIPA and approved by Privacy and Legal Office at ICES.

### 2.2 Data sources

ICES invested in health informatics for population health and health services research and housed individual-level high-quality health administrative databases on publicly funded services, including outpatient and inpatient visits (ED visits or hospitalizations) within Ontario (21). These databases are regularly updated and validated for accuracy (22, 23).^1^ The Registered Persons Database (RPDB) contains data on vital statistics, demographics and residence location. The Ontario Health Insurance Plan (OHIP) database captures 95% of physician billing. The National Ambulatory Care Reporting System Database (NACRS) records ED visits. The Discharge Abstract Database (DAD) records hospitalizations, and the Canadian Census includes neighborhood socioeconomic details. The COVID-19 Integrated Testing Dataset includes all available COVID-19 diagnostic laboratory results in Ontario. In Ontario, Canada, physician billing codes in response to COVID-19 were effective since March 14, 2020, and extended until September 30, 2022 (24). These databases were linked using unique encoded identifiers. Please see Supplementary Table S1 for details on the ICES databases used.

### 2.3 Population and setting

We adopted an open-cohort design where all adult Ontario residents (18 years and older) prior to and during the COVID-19 pandemic (January 2016–March 2021) who were alive at the beginning of each month were considered for inclusion. We excluded individuals younger than 18 years old, older than 105 years old, those who are not Ontario residents and with missing information on sex. The open cohort design allows individuals to enter the cohort as they age or leave the cohort when they die or move out of the province compared to a closed cohort with a fixed membership when after it is defined and follow-up begins, no one can be added to a cohort. We selected an open- vs. a closed-cohort study design to represent a real-world situation. We considered ***March 17, 2020,*** when the state of emergency was declared in Ontario (25), as the start of the pandemic. Follow-up continued until ***March 31, 2021***.

### 2.4 Time frame definitions

To avoid arbitrarily chosen categorization of the main period of interest (first year of the pandemic), observed versus projected rates with 95% confidence intervals (CIs) were visualized monthly. Only for comparisons, the main period of interest was divided into four time periods (26): (i) ***pre-COVID*-*19***: January–February 2020; (ii) ***Wave I***: March–May 2020; (iii) ***Summer Lull***: June–August 2020; and (iv) ***Wave II***: September 2020–March 2021.

On ***March 15, 2020***, the Ministry of Health in Ontario requested to ramp down non-emergent clinical activities to liberate health system capacity (27). Effective ***March 14, 2020***, temporary billing codes were introduced to allow remote outpatient sleep clinic visits to continue. After an initial increase in the number of COVID-19 cases in March–May 2020 (***Wave I***), a reduction was noted during June–August 2020 (***Summer Lull***), with a steep increase since September 2020 (***Wave II***) (28).

### 2.5 Outcomes

The primary outcomes were outpatient visits, ED visits, and hospitalizations, with the most responsible diagnosis for ACSCs considered separately (see Supplementary Table S2 for definition details) (29, 30). Outpatient visits were categorized by: (i) combined (virtual or in-person) primary care visits, (ii) combined (virtual or in-person) visits from a specialist with a relevant specialty (respirologist, cardiologist, endocrinologist, neurologist, internal medicine), and (ii) virtual outpatient visits. In Ontario, physician billing codes in response to the COVID-19 pandemic, including virtual visits (phone or video), were implemented on March 14, 2020, and extended until December 1, 2022 (24).

These outcomes were chosen because they reflect the acute and ambulatory care people receive for some of the most prevalent chronic diseases. We also wanted to assess how outcomes changed relative to each other to obtain a more fulsome understanding of the impact of the pandemic.

### 2.6 Statistical analyses

We used a similar analytic approach from our previous study of combined ACSCs patterns (12). Briefly, we aggregated data monthly to create a 63-period time series from January 2016 to March 2021 and calculated monthly rates as the number of events per 100,000 people. Auto-regressive integrated moving-average (ARIMA) modelling was used to calculate projected outcome rates based on observed monthly rates from similar periods in previous years (January 2016–December 2019). Specifically, to account for underlying trends and seasonality, ARIMA models regress a series of current observed rates on past values, fitting autoregressive and moving-average terms (31). We used SAS software’s adaption of the United States Census Bureau’s X-13ARIMA-SEATS program (X13) (32, 33), which also includes an automated model selection procedure (34). The selection procedure uses multiple algorithms to select ARIMA terms and the best fitting model using the Bayesian Information Criterion (32). Please see more details in the Data Supplement (Supplementary Text S1; Supplementary Figure S1).

The ARIMA procedure was chosen to allow for an automated model specification that would create projections taking into account secular and seasonal trends. We could then easily compare observed rates to projected rates and their 95% confidence intervals to determine how pandemic trends differed from what would be expected based on historic trends.

There is no definitive guidance on how many time points are required to apply ARIMA modelling (35). A minimum of 50 time points has been recommended (31), but this has no empirical basis and has not been tested formally. In uncomplicated cases, ARIMA can perform satisfactorily with short time series, as long as there are enough time points to estimate all parameters on the ARIMA model (36). In the presence of seasonality, there should be enough time points to identify the seasonal effects and to account for seasonal differencing. Usually, at least 4–5 years of data is recommended to forecast for 1–2 years, which was implemented in our study. More historical data also allows for greater flexibility in ARIMA model parameter selection as higher order models require more observations (36).

For each outcome, we used the best fitting model identified using the selection procedure described above to project monthly rates for 13 months following February 2020. While comparing observed vs. projected rates, we considered observed rates outside the projected 95% CIs to be significantly different (37). We graphically presented comparisons between observed and projected monthly rates as a time series. Mean rates across the four time periods were presented in tabular form.

All data analyses were performed using SAS (version 9.4 using SAS Enterprise guide version 7.15.3) following Ontario privacy standards in the secure environment at ICES.

#### 2.6.1 Subgroup analysis

We stratified analyses by sex and age group (18–24, 25–34, 35–49, 50–64, and 65 years and older).

## 3 Results

In March 2020, there were 12,211,898 adults in Ontario: 51.1% females and 21.9% 65 years and older.

Variations in rates and rate ratios of outpatient visits, ED visits, and hospitalizations compared to similar periods in previous years were found between ACSC, as presented in Tables 1–4 and Supplementary Tables S4–S9. Rates of virtual outpatient visits were significantly elevated during the first year of the pandemic for all ACSCs (Tables 1, 3).

**Table 1:** Monthly crude rates of outpatient visits for the separate Ambulatory Care Sensitive Conditions (ACSCs) during the first year of the COVID-19 pandemic with the crude rate ratios (RR) and confidence intervals (CI) to compare to similar periods in previous years.

Outcomes	2017–19	Pre-COVID 2020	RR (95%CI)	2017–19	Wave I 2020	RR (95%CI)	2017–19	Summer Lull 2020	RR (95%CI)	2017–19	Wave II 2020–21	RR (95%CI)
	January–February			March–May			June–August			September–March		
	Monthly rates per 100,000 people			Monthly rates per 100,000 people			Monthly rates per 100,000 people			Monthly rates per 100,000 people		
**Chronic obstructive pulmonary disease (COPD)**												
Primary care	160.09	143.80	0.90 (0.74–1.09)	159.29	126.40	**0.79** **(0.71–0.89)**	136.38	105.73	**0.78** **(0.70–0.86)**	150.41	112.55	**0.75** **(0.68–0.82)**
Relevant specialty	68.45	70.19	1.03 (0.92–1.15)	78.08	66.77	**0.86** **(0.78–0.94)**	70.72	68.40	0.97 (0.90–1.04)	71.75	78.13	1.09 (1.00–1.19)
Virtual, total	1.75	2.71	1.55 (0.99–2.42)	2.06	133.27	**64.82** **(43.36–96.92)**	1.97	124.67	**63.42** **(46.38–86.72)**	6.33	129.20	**20.42** **(8.16–51.10)**
**Asthma**												
Primary care	213.10	202.20	0.95 (0.76–1.19)	207.87	261.25	**1.26** **(1.08–1.46)**	183.35	187.04	1.02 (0.92–1.13)	207.82	184.68	0.89 (0.79–1.00)
Relevant specialty	73.34	80.50	1.10 (0.96–1.25)	82.41	82.41	1.00 (0.90–1.11)	75.63	85.54	**1.13** **(1.05–1.22)**	77.72	95.39	**1.23** **(1.13–1.33)**
Virtual, total	2.03	4.28	**2.11** **(1.39–3.19)**	2.34	266.99	**113.88** **(75.91–170.85)**	2.55	221.10	**86.76** **(57.52–130.87)**	12.68	217.24	**17.13** **(5.77–50.82)**
**Angina**												
Primary care	40.04	35.09	0.88 (0.75–1.03)	43.55	32.70	**0.75** **(0.67–0.84)**	39.59	33.80	**0.85** **(0.77–0.95)**	38.92	33.42	**0.86** **(0.78–0.94)**
Relevant specialty	51.27	52.87	1.03 (0.81–1.32)	58.85	47.52	**0.81** **(0.75–0.87)**	58.08	52.84	**0.91** **(0.84–0.98)**	55.73	55.94	1.00 (0.89–1.13)
Virtual, total	0.39	0.45	1.15 (0.26–5.16)	0.36	47.58	**132.69** **(42.52–414.12)**	0.20	55.71	**279.61 (110.69–706.36)**	1.55	55.42	**35.84** **(10.79–119.03)**
**Congestive heart failure (CHF)**												
Primary care	92.13	95.53	1.04 (0.93–1.16)	101.61	100.17	0.99 (0.93–1.04)	95.55	104.48	**1.09** **(1.02–1.17)**	94.02	104.75	**1.11** **(1.05–1.18)**
Relevant specialty	84.06	92.09	1.10 (0.98–1.23)	96.05	87.33	**0.91** **(0.84–0.98)**	88.91	97.59	**1.10** **(1.02–1.18)**	90.23	101.55	**1.13** **(1.05–1.21)**
Virtual, total	0.53	0.68	**1.29** **(1.08–1.55)**	0.50	110.97	**221.55 (147.33–333.18)**	0.45	112.43	**249.52 (168.60–369.28)**	3.31	108.04	**32.67** **(10.10–105.66)**
**Diabetes**												
Primary care	1510.50	1611.26	1.07 (0.95–1.19)	1676.76	1335.98	**0.80** **(0.73–0.87)**	1585.48	1545.17	0.97 (0.92–1.03)	1596.48	1659.51	1.04 (0.97–1.12)
Relevant specialty	333.58	369.86	**1.11** **(1.02–1.21)**	373.44	385.52	1.03 (0.95–1.12)	343.01	406.69	1.19 (1.12–1.26)	353.61	421.41	1.19 (1.10–1.29)
Virtual, total	5.71	13.67	**2.39** **(1.45–3.95)**	6.68	1128.21	**168.96 (105.42–270.80)**	7.22	1322.19	**183.11 (117.05–286.46)**	39.07	1333.93	**34.10 (11.11–104.62)**
**Hypertension**												
Primary care	1681.99	1695.46	1.01 (0.90–1.13)	1851.64	1487.88	**0.80** **(0.75–0.86)**	1716.44	1533.65	**0.89** **(0.83–0.96)**	1744.27	1660.16	0.95 (0.88–1.02)
Relevant specialty	111.20	110.88	1.00 (0.89–1.11)	126.98	95.81	**0.75** **(0.70–0.81)**	112.29	101.95	**0.91** **(0.85–0.97)**	113.98	119.33	1.05 (0.97–1.13)
Virtual, total	5.35	14.48	**2.71** **(1.30–5.65)**	6.28	1053.13	**167.75** **(94.98–296.26)**	7.46	1117.46	**149.84 (94.34–238.00)**	38.18	1119.79	**29.33** **(9.69–88.73)**
**Epilepsy**												
Primary care	27.23	25.34	0.93 (0.83–1.04)	28.05	25.68	**0.92** **(0.87–0.96)**	27.19	29.25	**1.08** **(1.01–1.14)**	26.61	29.63	**1.11** **(1.04–1.19)**
Relevant specialty	43.40	45.60	1.05 (0.95–1.16)	47.57	46.73	0.98 (0.89–1.08)	43.86	48.38	**1.10** **(1.04–1.17)**	44.66	53.26	**1.19** **(1.10–1.29)**
Virtual, total	1.06	1.55	**1.47** **(1.13–1.90)**	1.12	52.26	**46.53** **(31.63–68.45)**	1.20	60.19	**50.29** **(40.36–62.67)**	2.49	63.33	**25.40 (12.20–52.87)**

In bold: statistically significant. CI, confidence intervals; ED, emergency department; RR, rate ratios.

**Table 2:** Monthly crude rates of hospitalizations and emergency department (ED) for the separate Ambulatory Care Sensitive Conditions (ACSCs) during the first year of the COVID-19 pandemic with the crude rate ratios (RR) and confidence intervals (CI) to compare to similar periods in previous years.

Outcomes	2017–19	Pre-COVID 2020	RR (95%CI)	2017–19	Wave I 2020	RR (95%CI)	2017–19	Summer Lull 2020	RR (95%CI)	2017–19	Wave II 2020–21	RR (95%CI)
	January–February			March–May			June–August			September–March		
	Monthly rates per 100,000 people			Monthly rates per 100,000 people			Monthly rates per 100,000 people			Monthly rates per 100,000 people		
**Hospitalizations for ACSCs**												
**COPD**	23.30	20.49	0.88 (0.70–1.10)	20.12	11.26	**0.56** **(0.48–0.65)**	15.47	9.78	**0.63** **(0.58–0.69)**	19.3	9.42	**0.49** **(0.43–0.56)**
**Asthma**	1.70	1.47	0.86 (0.70–1.05)	1.66	0.90	**0.54** **(0.43–0.68)**	1.23	0.66	**0.54** **(0.44–0.65)**	1.55	0.70	**0.45** **(0.39–0.52)**
**Angina**	3.03	2.53	0.84 (0.74–0.95)	3.13	1.87	**0.60** **(0.53–0.67)**	3.04	2.48	**0.81** **(0.75–0.89)**	2.90	2.23	**0.77** **(0.70–0.85)**
**CHF**	18.62	19.51	1.05 (0.95–1.15)	20.15	14.20	**0.70** **(0.65–0.81)**	17.29	16.18	**0.94** **(0.91–0.96)**	18.61	16.09	**0.86** **(0.82–0.92)**
**Diabetes**	5.02	5.38	1.07 (0.94–1.23)	5.02	4.36	**0.87** **(0.82–0.92)**	4.84	5.08	1.05 (0.98–1.13)	5.09	4.80	0.94 (0.89–1.00)
**Hypertension**	1.57	1.66	1.06 (0.92–1.22)	1.68	1.15	**0.68** **(0.58–0.80)**	1.51	1.48	0.98 (0.85–1.12)	1.62	1.64	1.01 (0.91–1.13)
**Epilepsy**	2.55	2.62	1.03 (0.90–1.17)	2.63	1.96	**0.74** **(0.65–0.81)**	2.69	2.43	**0.91** **(0.84–0.98)**	2.60	2.44	0.94 (0.88–1.01)
**Emergency department (ED) visits for ACSCs**												
**COPD**	45.21	39.80	0.88 (0.71–1.10)	40.37	21.93	**0.54** **(0.46–0.64)**	32.13	19.04	**0.59** **(0.55–0.64)**	39.14	17.82	**0.46** **(0.41–0.51)**
**Asthma**	17.20	15.24	0.89 (0.72–1.10)	17.03	10.69	**0.63** **(0.49–0.80)**	13.96	7.47	**0.54** **(0.47–0.60)**	16.82	7.58	**0.45** **(0.39–0.52)**
**Angina**	8.80	7.73	**0.88** **(0.80–0.97)**	9.07	6.17	**0.68** **(0.62–0.74)**	8.56	7.06	**0.83** **(0.78–0.88)**	8.51	6.71	**0.79** **(0.73–0.85)**
**CHF**	24.54	24.49	1.00 (0.88–1.13)	26.47	17.07	**0.64** **(0.58–0.71)**	22.96	20.01	**0.87** **(0.84–0.90)**	24.07	19.61	**0.81** **(0.76–0.87)**
**Diabetes**	13.67	13.88	1.02 (0.90–1.14)	14.10	9.79	**0.69** **(0.63–0.76)**	13.72	12.37	**0.90** **(0.86–0.94)**	13.39	11.51	**0.86** **(0.81–0.91)**
**Hypertension**	23.79	26.54	**1.12** **(1.04–1.20)**	24.80	16.87	**0.68** **(0.59–0.78)**	20.52	19.12	0.93 (0.85–1.02)	24.34	23.33	0.96 (0.89–1.03)
**Epilepsy**	6.70	6.69	1.00 (0.92–1.09)	7.15	5.32	**0.74** **(0.68–0.82)**	7.41	6.08	**0.82** **(0.76–0.88)**	6.92	6.00	**0.87** **(0.83–0.91)**

In bold: statistically significant. CI, confidence intervals; ED, emergency department; RR, rate ratios.

**Table 3:** Observed and projected monthly rates and 95% confidence intervals (CI) estimated by ARIMA Models for outpatient visits for the separate Ambulatory Care Sensitive Conditions (ACSCs) during the first year of the COVID-19 pandemic: rates were calculated as the number of events per 100,000 people at risk.

Outcomes	Observed	Projected (95% CI)	Observed	Projected (95% CI)	Observed	Projected (95% CI)	Observed	Projected (95% CI)
	Pre-COVID		Wave I		Summer Lull		Wave II	
	January–February 2020		March–May 2020		June–August 2020		September 2020–March 2021	
**COPD**								
ACSC specialty	70.19	65.99 (60.14–72.41)	**66.77**	80.35 (73.10–88.31)	68.40	71.35 (64.24–79.25)	78.13	72.49 (64.60–81.35)
Primary care	143.80	139.73 (126.88–153.87)	**126.40**	142.86 (129.61–157.48)	**105.73**	121.05 (109.64–133.65)	**112.55**	133.96 (120.92–148.41)
Virtual visit	2.71	2.78 (2.35–3.21)	**133.27**	3.13 (2.66–3.60)	**124.67**	2.96 (2.38–3.54)	**129.20**	3.18 (2.42–3.93)
**Asthma**								
ACSC specialty	80.50	74.21 (66.25–83.13)	82.41	84.11 (75.08–94.22)	85.54	76.78 (68.55–86.01)	**95.39**	79.54 (70.66–89.54)
Primary care	202.20	189.90 (168.13–214.49)	**261.25**	196.79 (173.13–223.69)	187.04	171.15 (149.96–195.32)	184.68	188.29 (163.85–216.44)
Virtual visits	4.28	4.57 (4.00–5.14)	**266.99**	4.30 (3.52–5.09)	**221.10**	4.56 (3.30–5.83)	**217.24**	4.80 (3.00–6.60)
**Angina**								
ACSC specialty	52.87	50.63 (42.42–58.84)	**47.52**	60.43 (51.97–68.89)	**52.84**	62.08 (52.91–71.25)	55.94	62.14 (51.34–72.93)
Primary care	35.09	33.82 (30.86–37.06)	**32.70**	36.54 (33.34–40.05)	33.80	33.15 (30.25–36.33)	33.42	33.05 (30.07–36.33)
Virtual visit	0.45	0.30 (0.06–1.38)	**47.58**	0.14 (0.03–0.80)	**55.71**	0.15 (0.02–1.03)	**55.42**	0.15 (0.02–1.03)
**CHF**								
ACSC specialty	92.09	86.87 (80.17–94.13)	**87.33**	103.80 (95.37–112.97)	97.59	94.68 (86.42–103.73)	101.55	97.53 (88.34–107.70)
Primary care	95.53	95.11 (88.53–102.18)	100.17	103.71 (96.54–111.43)	104.48	97.61 (90.85–104.87)	**104.75**	96.42 (89.40–104.00)
Virtual visit	0.68	0.45 (0.08–0.81)	**110.97**	0.45 (0.08–0.81)	**112.43**	0.45 (0.08–0.81)	**108.04**	0.45 (0.08–0.81)
**Diabetes**								
ACSC specialty	369.86	358.38 (335.34–382.99)	385.52	404.39 (374.13–437.13)	**406.69**	370.98 (340.49–404.20)	421.41	384.98 (351.37–421.84)
Primary care	**1611.26**	1,470.68 (1,366.01-1,583.37)	**1335.98**	1,675.04 (1,554.45-1,804.99)	1545.17	1,580.98 (1,466.51-1,704.38)	1659.51	1,626.34 (1,502.23-1,760.89)
Virtual visit	13.67	12.66 (11.33–14.00)	**1128.21**	13.02 (11.67–14.38)	**1322.19**	14.11 (12.13–16.08)	**1333.93**	15.45 (12.34–18.55)
**Hypertension**								
ACSC specialty	110.88	104.71 (95.50–114.80)	**95.81**	124.65 (113.44–136.97)	101.95	109.09 (98.19–121.21)	119.33	110.77 (97.96–125.30)
Primary care	1695.46	1,635.88 (1,514.66-1,766.80)	**1487.88**	1,826.44 (1,690.92-1,972.82)	1533.65	1,613.51 (1,470.97-1,769.86)	1660.16	1,699.95 (1,543.27-1,872.75)
Virtual visit	14.48	13.50 (12.45–14.54)	**1053.13**	15.57 (14.22–16.91)	**1117.46**	16.36 (14.14–18.58)	**1119.79**	18.03 (14.76–21.30)
**Epilepsy**								
ACSC specialty	45.60	43.70 (39.80–47.97)	46.73	48.70 (44.26–53.58)	48.38	45.21 (41.05–49.79)	**53.26**	45.62 (41.21–50.52)
Primary care	25.34	24.05 (22.23–26.03)	25.68	26.03 (23.96–28.27)	**29.25**	24.93 (22.75–27.32)	**29.63**	24.65 (22.09–27.50)
Virtual visit	1.55	1.38 (1.14–1.63)	**52.26**	1.45 (1.18–1.72)	**60.19**	1.46 (1.14–1.78)	**63.33**	1.44 (1.02–1.85)

Similar periods in previous years (2016–2019) were used to calculate projected rates. In bold: observed rates outside the projected 95% confidence intervals of projected rates were considered as significantly different.

**Table 4:** Observed and projected monthly rates and 95% confidence intervals (CI) estimated by ARIMA Models for hospitalizations and emergency department (ED) for the separate Ambulatory Care Sensitive Conditions (ACSCs) during the first year of the COVID-19 pandemic: rates were calculated as the number of events per 100,000 people at risk.

Outcomes	Observed	Projected (95% CI)	Observed	Projected (95% CI)	Observed	Projected (95% CI)	Observed	Projected (95% CI)
	Pre-COVID		Wave I		Summer Lull		Wave II	
	January–February 2020		March–May 2020		June–August 2020		September 2020–March 2021	
**ED visit**								
Asthma	15.24	16.12 (13.65–19.04)	**10.69**	15.51 (13.04–18.43)	**7.47**	12.72 (10.66–15.18)	**7.58**	15.28 (12.77–18.30)
COPD	39.80	40.19 (34.89–46.31)	**21.93**	39.17 (32.66–47.00)	**19.04**	31.63 (25.89–38.65)	**17.82**	37.39 (30.42–45.98)
Angina	7.73	7.69 (7.05–8.38)	**6.17**	7.97 (7.29–8.71)	7.06	7.55 (6.84–8.32)	**6.71**	7.65 (6.84–8.55)
CHF	24.49	25.39 (23.33–27.44)	**17.07**	27.29 (25.23–29.35)	**20.01**	23.35 (21.29–25.41)	**19.61**	24.84 (22.76–26.92)
Hypertension	26.54	25.45 (23.21–27.68)	**16.87**	26.18 (23.89–28.47)	**19.12**	22.08 (19.59–24.58)	23.33	26.19 (23.28–29.10)
Diabetes	13.88	13.53 (12.84–14.26)	**9.79**	13.91 (13.17–14.70)	**12.37**	13.43 (12.66–14.25)	**11.51**	13.41 (12.64–14.24)
Epilepsy	6.69	6.51 (6.04–7.01)	**5.32**	6.96 (6.46–7.50)	**6.08**	7.11 (6.60–7.66)	**6.00**	6.73 (6.24–7.26)
**Hospitalizations**								
Asthma	1.47	1.68 (1.32–2.14)	**0.90**	1.69 (1.33–2.15)	**0.66**	1.21 (0.95–1.54)	**0.70**	1.51 (1.18–1.93)
COPD	20.49	20.79 (18.21–23.73)	**11.26**	19.52 (16.50–23.09)	**9.78**	15.21 (12.64–18.30)	**9.42**	18.43 (15.22–22.32)
Angina	2.53	2.64 (2.31–3.02)	**1.87**	2.77 (2.42–3.17)	2.48	2.60 (2.27–2.98)	**2.23**	2.58 (2.25–2.96)
CHF	19.51	19.50 (18.40–20.68)	**14.20**	20.87 (19.64–22.18)	**16.18**	17.94 (16.85–19.11)	**16.09**	19.68 (18.46–20.99)
Hypertension	1.66	1.61 (1.30–1.99)	**1.15**	1.58 (1.28–1.96)	1.48	1.59 (1.28–1.97)	1.64	1.59 (1.28–1.98)
Diabetes	5.38	5.10 (4.62–5.59)	**4.36**	5.07 (4.57–5.57)	5.08	4.89 (4.38–5.41)	4.80	5.08 (4.56–5.60)
Epilepsy	2.62	2.54 (2.23–2.89)	**1.96**	2.60 (2.28–2.97)	2.43	2.68 (2.35–3.06)	2.44	2.59 (2.27–2.97)

Similar periods in previous years (2016–2019) were used to calculate projected rates. In bold: observed rates outside the projected 95% confidence intervals of projected rates were considered as significantly different.

### 3.1 Respiratory conditions

#### 3.1.1 Asthma

**Figure 1:**
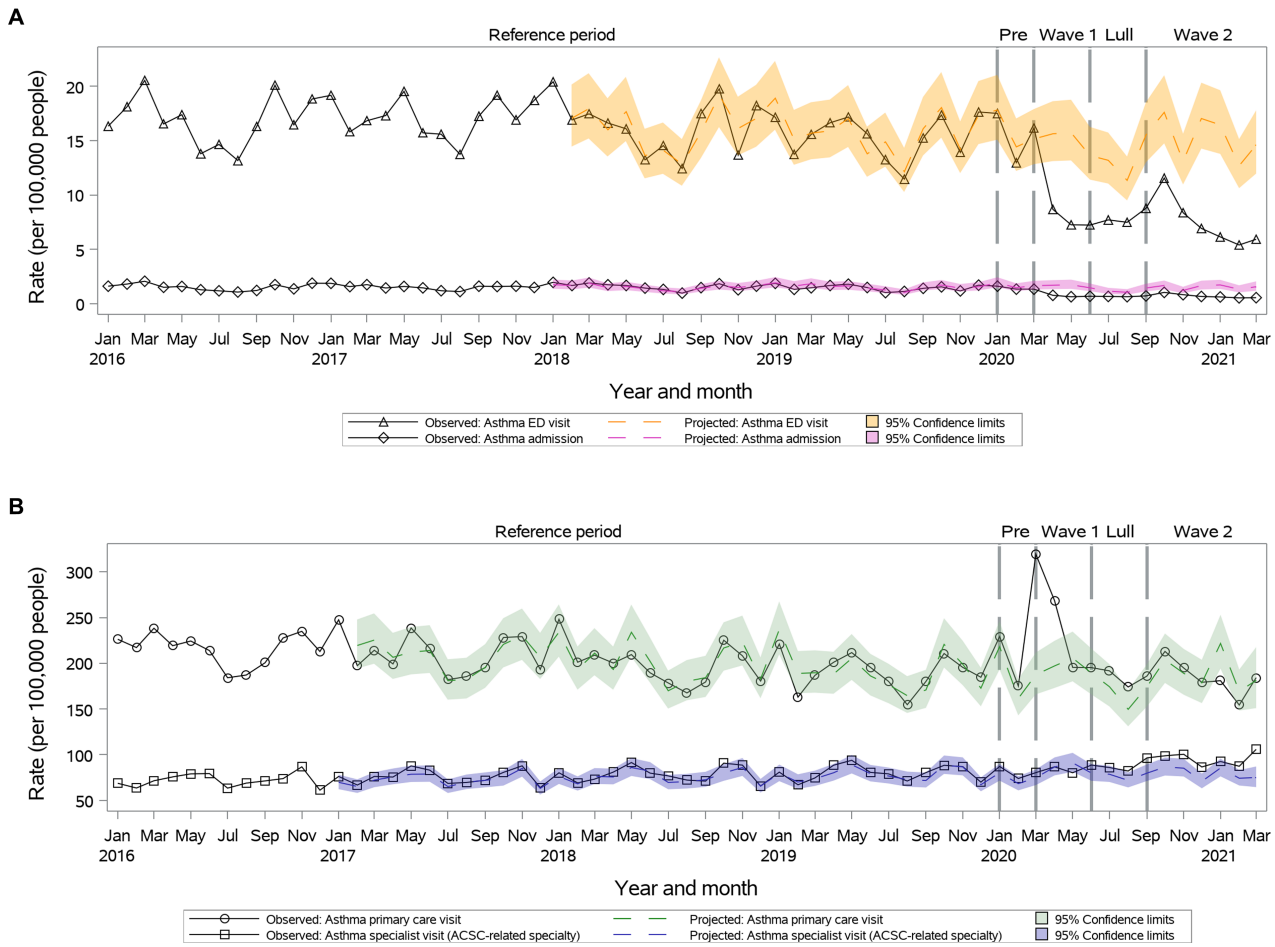
Observed versus projected monthly rates per 100,000 people at-risk for **asthma**: **(A)** Acute care visits: emergency department (ED) visits or hospitalizations; **(B)** Outpatient visits: primary care or relevant specialty visits; ambulatory-care-sensitive conditions (ACSCs).

Primary care visit rates were higher than projected during ***Wave I*** (261.3 vs. 196.8, 95% CI: 173.1–223.7), but returned to projected during ***Summer Lull*** and ***Wave II*** (Figure 1). Specialty visit rates remained as projected during ***Wave I*** and ***Summer Lull*** and were higher than projected during ***Wave II*** (95.4 vs. 79.5, 95% CI: 70.7–89.5) (Figure 1). We observed significant reductions in ED visits and hospitalizations vs. projected rates during the first year of the pandemic (Figure 1).

During ***Wave I***, primary care visits were above projected for all, but individuals 65 years or older. Similarly, specialist care during ***Wave II*** remained above projected for all, but individuals 65 years or older. Age and sex did not influence inpatient visit trends.

#### 3.1.2 COPD

**Figure 2:**
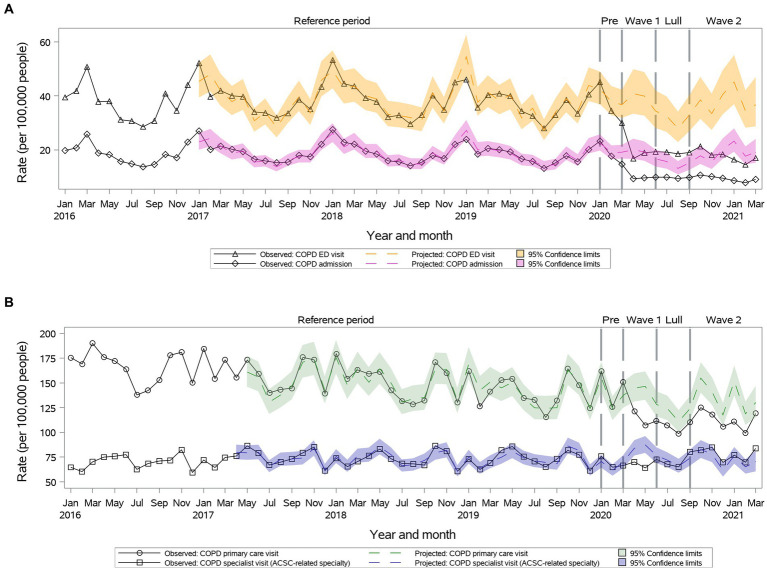
Observed versus projected monthly rates per 100,000 people at-risk for **chronic obstructive pulmonary disease (COPD)**: **(A)** Acute care visits: emergency department (ED) visits or hospitalizations; **(B)** Outpatient visits: primary care or relevant specialty visits; ambulatory-care-sensitive conditions (ACSCs).

We observed a significant reduction in primary care visit rates during the first year of the pandemic (112.6 vs. 134.0, 95% CI: 120.9–148.4 during ***Wave II***) (Figure 2). Observed speciality visit rates were lower than projected during ***Wave I***, but then returned to projected during ***Summer Lull*** and ***Wave II*** (Figure 2). Further, during the first year of the pandemic, we observed significant reductions in ED visits and hospitalizations observed vs. projected rates (Figure 2).

Age and sex did not influence outpatient and inpatient visit trends.

### 3.2 Cardiometabolic conditions

#### 3.2.1 Angina

**Figure 3:**
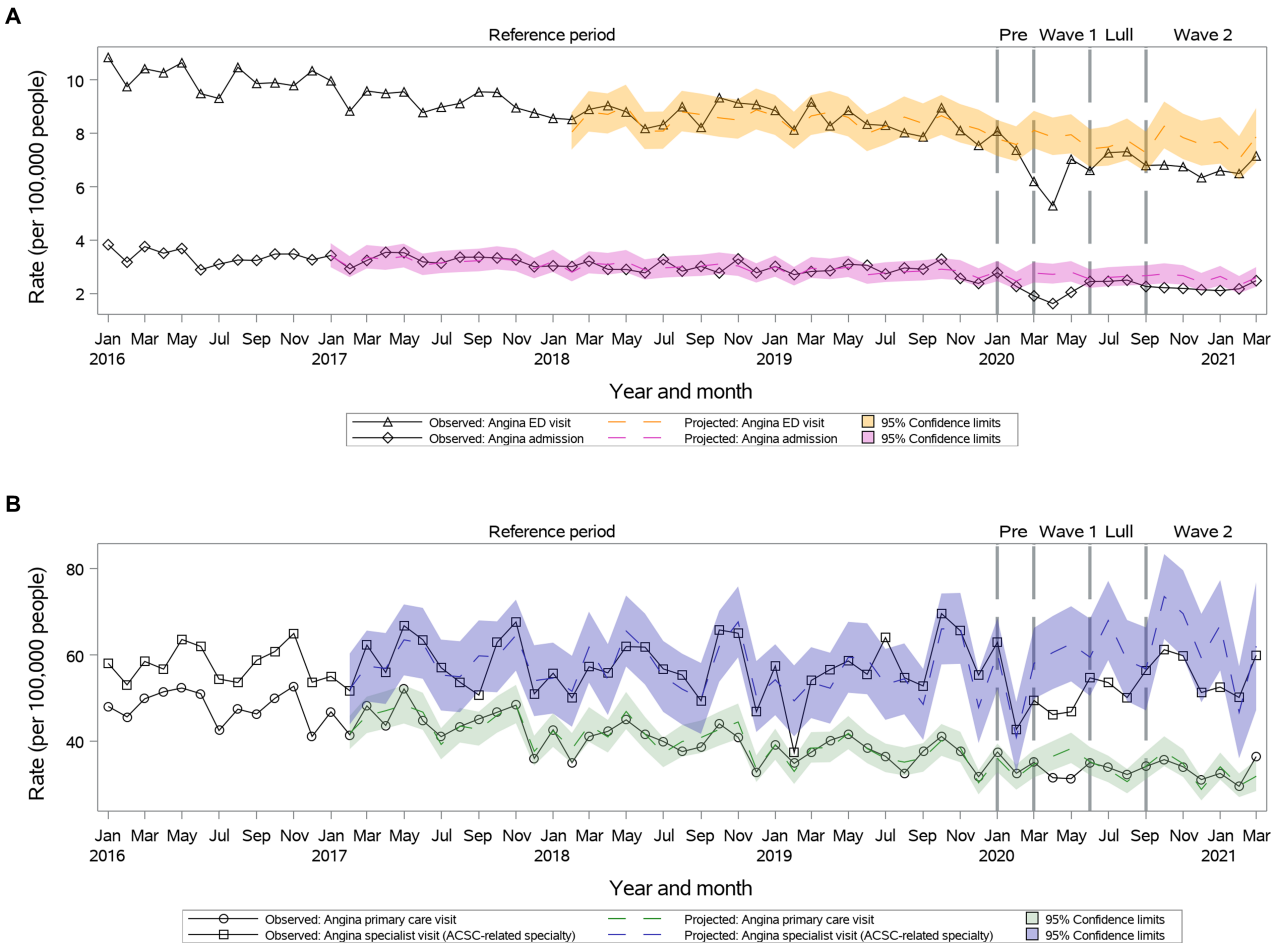
Observed versus projected monthly rates per 100,000 people at-risk for **angina**: **(A)** Acute care visits: emergency department (ED) visits or hospitalizations; **(B)** Outpatient visits: primary care or relevant specialty visits; ambulatory-care-sensitive conditions (ACSCs).

Primary care visit rates were lower than projected during ***Wave I***, but then returned to projected during ***Summer Lull*** and ***Wave II*** (33.4 vs. 33.1, 95% CI: 30.1–36.3 during ***Wave II***) (Figure 3). Specialty visit rates were lower than projected during ***Wave I*** and ***Summer Lull*** and returned to projected during ***Wave II*** (55.9 vs. 62.1, 95% CI: 51.3–72.9) (Figure 3). We observed significant reductions in ED visits and hospitalizations rates vs. projected rates during ***Waves I*** and ***II***, which returned to projected during ***Summer Lull*** (Figure 3).

Primary and specialty care visits, ED visits and hospitalizations rates remained as projected during the first year of the pandemic for individuals 18–34 years old. In females, hospitalizations remained below projected during the first year of the pandemic. In men, hospitalizations returned to projected during ***Summer Lull*** and ***Wave II***.

#### 3.2.2 CHF

**Figure 4:**
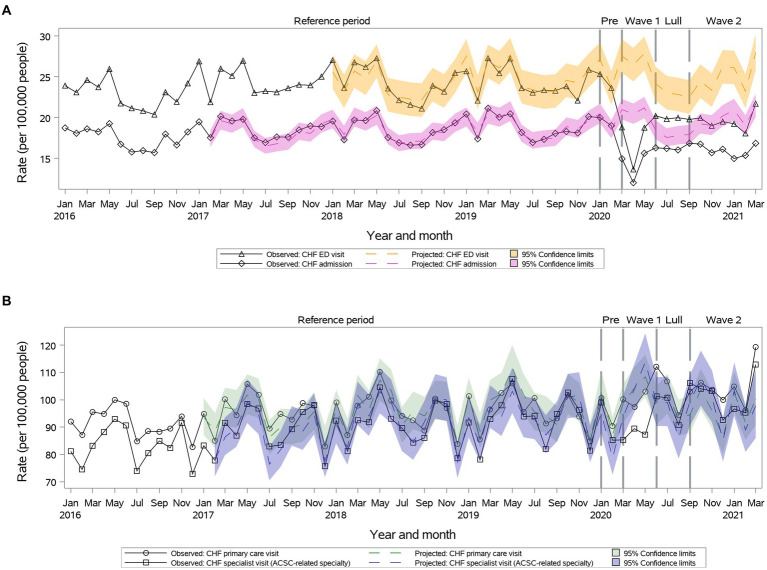
Observed versus projected monthly rates per 100,000 people at-risk for **congestive heart failure (CHF)**: **(A)** Acute care visits: emergency department (ED) visits or hospitalizations; **(B)** Outpatient visits: primary care or relevant specialty visits; ambulatory-care-sensitive conditions (ACSCs).

Primary outpatient visit rates remained as projected during ***Wave I*** and ***Summer Lull*** and became significantly higher than projected during ***Wave II*** (104.8 vs. 96.4, 95% CI: 89.4–104.0) (Figure 4). Specialty visits were reduced during ***Wave I*** and then returned to the projected during ***Summer Lull*** and ***Wave II*** (Figure 4). We observed significant reductions in ED visits and hospitalizations rates vs. projected rates during the first year of the pandemic (Figure 4).

Primary care visit rates remained as projected during the first year of the pandemic for individuals 18–34 and older than 65 years old. Specialty care visit rates remained as projected during the first year of the pandemic for individuals 18–34 years old. The ED visits and hospitalizations remained as projected for individuals 18–49 years old.

#### 3.2.3 Hypertension

**Figure 5:**
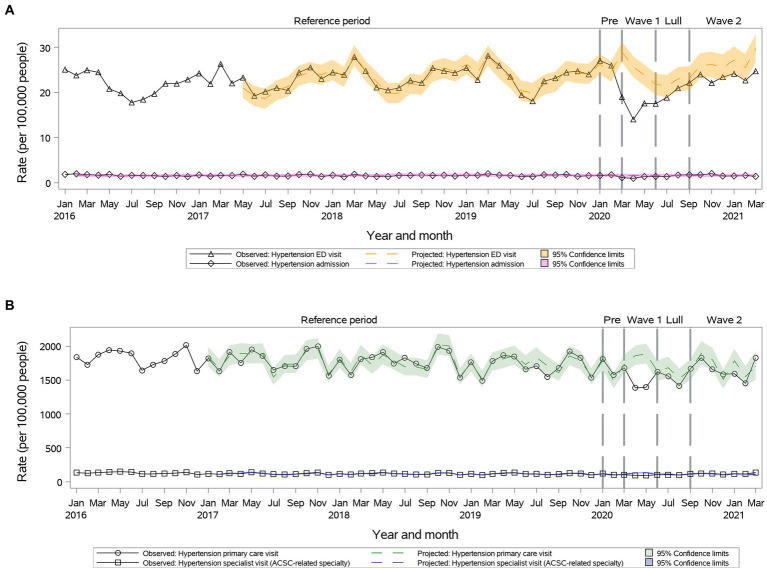
Observed versus projected monthly rates per 100,000 people at-risk for **hypertension**: **(A)** Acute care visits: emergency department (ED) visits or hospitalizations; **(B)** Outpatient visits: primary care or relevant specialty visits; ambulatory-care-sensitive conditions (ACSCs).

Primary care and specialty visits were reduced during ***Wave I*** and returned to projected during ***Summer Lull*** and ***Wave II*** (Figure 5). There was a significant decrease in ED visit rates during ***Wave I*** and ***Summer Lull***, which returned to the projected during ***Wave II*** (23.3 vs. 26.2, 95% CI: 23.3–29.1) (Figure 5). We observed a significant reduction in hospitalizations rates only during ***Wave I***, which returned to the projected during ***Summer Lull*** and ***Wave II*** (Figure 5).

Primary care pattern was similar across age and sex subgroups. Specialty visit rates remained as projected for the entire year in individuals 25–34 years old. Hospitalization rates remained as projected in individuals 18–64 years old.

#### 3.2.4 Diabetes

**Figure 6:**
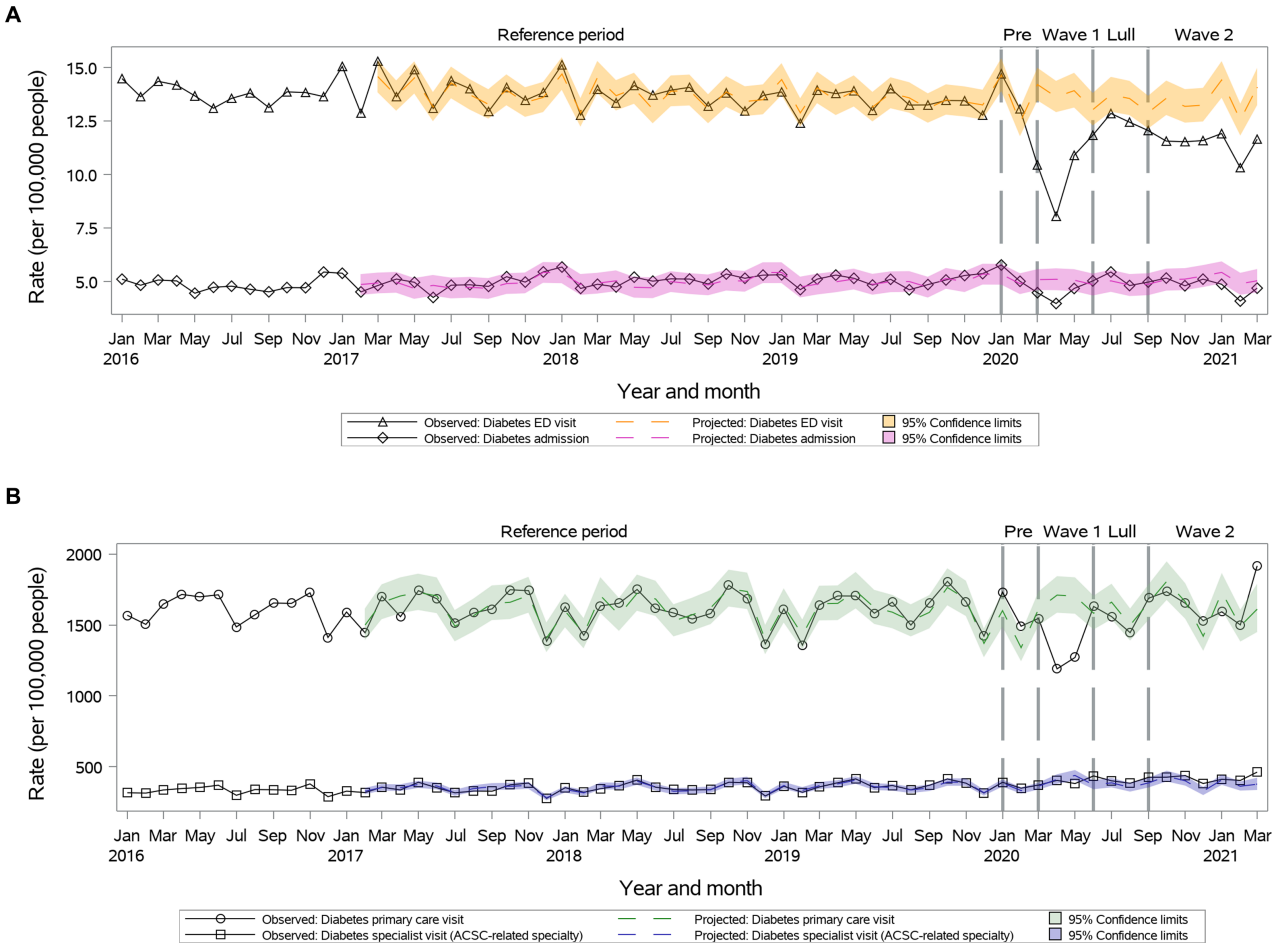
Observed versus projected monthly rates per 100,000 people at-risk for **diabetes**: **(A)** Acute care visits: emergency department (ED) visits or hospitalizations; **(B)** Outpatient visits: primary care or relevant specialty visits; ambulatory-care-sensitive conditions (ACSCs).

During ***Wave I***, we observed a significant reduction in primary care visit rates (1,336.0 vs. 1,675.0, 95% CI: 1,554.5-1,805.0); rates returned to the projected during ***Summer Lull*** and ***Wave II*** (Figure 6). The specialty visit rates were reduced compared to the projected during ***Wave I*** and then returned to the projected (Figure 6). We observed a significant reduction in ED visit rates during the first year of the pandemic (Figure 6). A significant reduction in hospitalization rates was observed only during only ***Wave I***, which returned to the projected during ***Summer Lull*** and ***Wave II*** (Figure 6).

Primary care rates increased above projected during ***Wave II*** in individuals 18–34 years old. Specialty visit rates increased above projected during ***Wave II*** in individuals 18–49 years old and females. Hospitalization rates remained as projected for individuals 35–64 years old for the entire year.

### 3.3 Epilepsy

**Figure 7:**
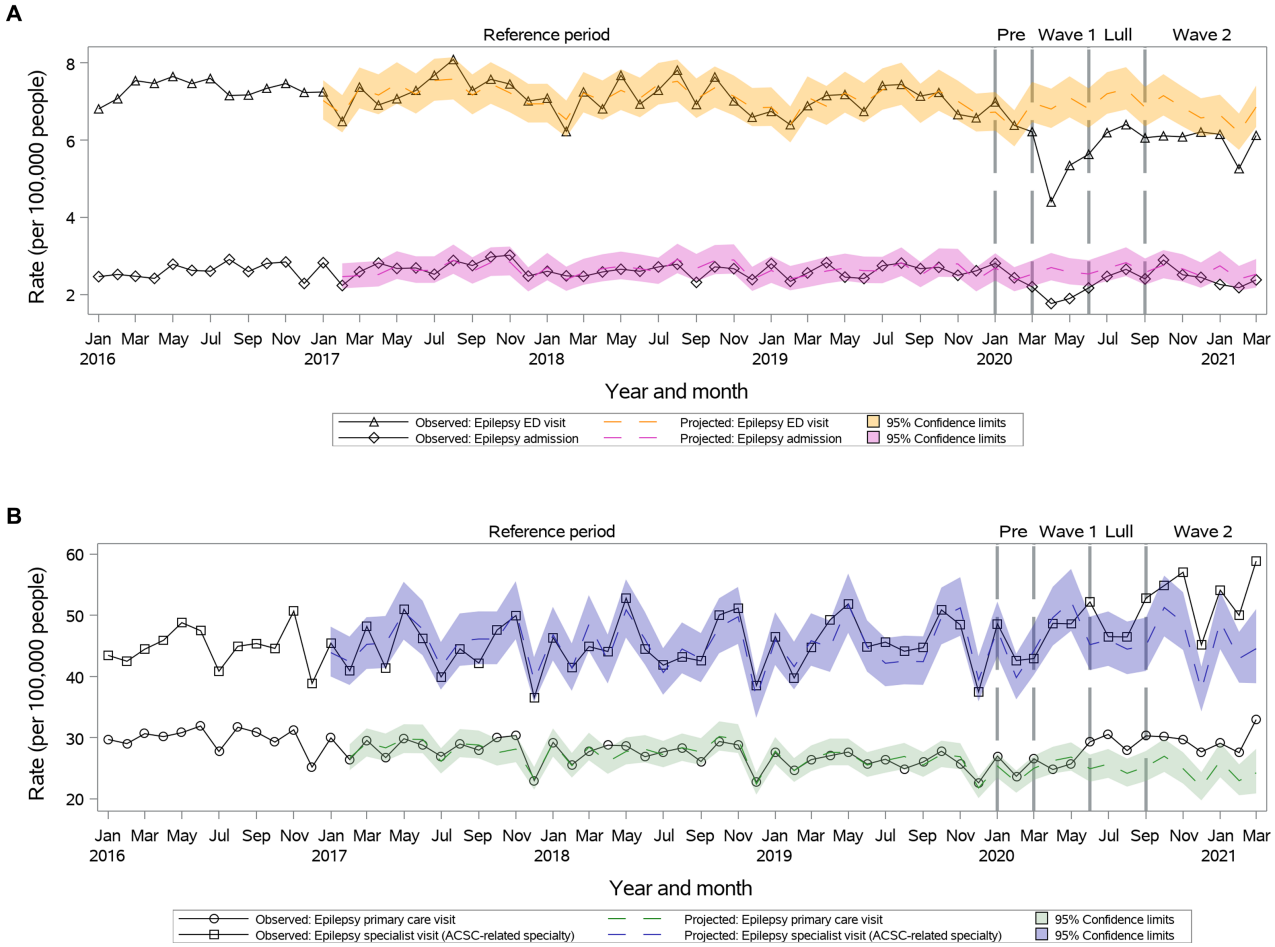
Observed versus projected monthly rates per 100,000 people at-risk for **epilepsy**: **(A)** Acute care visits: emergency department (ED) visits or hospitalizations; **(B)** Outpatient visits: primary care or relevant specialty visits; ambulatory-care-sensitive conditions (ACSCs).

While outpatient primary care visits remained as projected during ***Wave I***, rates increased significantly above the projected during ***Summer Lull*** and ***Wave II*** (29.6 vs. 24.7, 95% CI: 22.1–27.5 during ***Wave II***) (Figure 7). Specialty visits remained as projected during ***Wave I*** and ***Summer Lull***, then increased significantly during ***Wave II*** (53.26 vs. 45.62, 95% CI: 41.21–50.52) (Figure 7). We observed a significant reduction in ED visit rates during the first year of the pandemic (Figure 7). A significant reduction in hospitalization rates was observed only during ***Wave I***, which returned to the projected during ***Summer Lull*** and ***Wave II*** (Figure 7).

Age and sex did not influence outpatient care visit rates. Hospitalization rates remained as projected among individuals 18–24 and 34–49 years old.

## 4 Discussion

We conducted a population study of trends in outpatient visits, ED visits, and hospitalizations in the first year of the COVID-19 pandemic and found notable variations for seven ACSCs. While initial reductions in outpatient visits resolved towards the end of the first year, they remained low for COPD. Encouragingly, these reductions did not result in more hospitalizations for any ASCS; in fact, hospitalizations were low for asthma, COPD, and CHF. To the best of our knowledge, ours is the first population study examining trends in all outpatient visits, ED visits, and hospitalizations for seven ACSCs. Its examination of seven ACSC conditions and their rates compared to previous years reveal significant disruption in the healthcare system lasting well beyond the initial shock at the beginning of the pandemic. Luckily this shock did not seem to lead to an overwhelming number of hospitalizations, and it is important to understand how catastrophe was avoided.

Regardless of outpatient visit trends, observed ED visits and hospitalizations did not exceed projected rates in post-pandemic periods. Rates of ED visits remained below projected during the first year of the pandemic for asthma, COPD, angina, diabetes, and epilepsy, and returned to projected for CHF and hypertension. Among the seven ACSCs, reductions in inpatient visits (both ED visits and hospitalizations) were most pronounced for COPD and asthma. CHF-related hospitalizations also consistently decreased during the pandemic, albeit to a lesser extent.

There are several possible explanations for why low rates of outpatient visits did not result in more ED visits and hospitalizations as would be expected of ACSCs. First, patients avoided the ED and hospital because they feared catching COVID-19 (1, 38–40). This is concerning because not seeking care may have resulted in them being admitted later with more advanced disease (41–43). Second, a focus on COVID-19 meant essential healthcare services were not available for non-COVID-19 conditions, thus limiting resources for ACSC patients (44–46). Third, lower rates of screening and diagnostic testing prevented the recognition of patients requiring hospitalization (47, 48). Fourth, there was a true decrease in need for ACSC hospitalizations because stay-at-home orders led to fewer circulating seasonal respiratory viruses, less air pollution, and patients having more time to focus on their health (1, 49, 50). In support of the latter, we demonstrated the most pronounced reductions in inpatient visits for COPD and asthma as a possible unintended benefit. In addition, an increase in adherence to medications for asthma and COPD was also noted this time (51–53). Fifth, low rates of outpatient visits may not have resulted in more ED visits and hospitalizations because virtual care was able to effectively replace in-person visits (1, 45, 54). Finally, it may not be one of these explanations, but many of them in combination. Future research on which of these explanations/strategies are most prominent is important to guide where resources are best placed for the next pandemic. Given the possible importance of respiratory viruses as a trigger for exacerbations of certain ACSCs, maybe more resources should be devoted to preventing the spread of respiratory infection through ventilation and vaccination as a public health intervention. Virtual care and stay-at-home orders are arguably effective strategies. Nonetheless, those strategies are greatly disruptive and have their own risks; therefore, more studies to learn how to refine these strategies should be done in anticipation of future pandemics or other disasters.

Our study was consistent with others that demonstrated reductions in outpatient care during the pandemic. For example, there was a significant decrease in diabetes-related outpatient care visits at the beginning of the pandemic, with a rebound in the later stages (55, 56), and an increase in telemedicine utilization (56). We found an increase in outpatient primary care visits for asthma in the first two months of the pandemic, which may be explained by an overlap in symptoms between asthma and COVID-19 and limited access to COVID-19 testing and specialists at the beginning of the pandemic. This was in contrast to existing literature, where a reduction in outpatient primary care visits for asthma was observed (57, 58). Our findings also agreed with studies demonstrating a decline in in-person medical visits for chronic cardiovascular conditions at the beginning of the pandemic, specifically reductions in hospitalizations and ED visits by 25–85% (16, 59–61), 40–80% (62, 63), and 16% (16) for angina, CHF, and hypertension, respectively. Inpatient and ED visits for COPD or asthma declined by approximately 40 and 80%, respectively, at the beginning of the pandemic (15, 64, 65). Similarly, epilepsy-related hospitalizations and ED visits were found to be significantly decreased (4–38%) during the early stages of the COVID-19 pandemic despite antiepileptic medication shortages and increased barriers to in-person tests such as EEG monitoring (9, 66–68). Again, public health restrictions and lock-down measures might have led to improved home management of epilepsy (67), which is supported by high patient satisfaction and lower no-show rates associated with telehealth solutions such as electronic seizure journals, mobile apps, and video conferencing (69).

Our study extends these previous findings by detailing the health system response, which is important for many reasons. First, we demonstrated that – despite the introduction of virtual care and adaptations – disruptions in the health care system and, specifically, the care provided for certain diseases such as COPD persisted until the end of the first year of the pandemic. Some may argue that this is the “new normal” and extends beyond the first year and/or reverberates in other ways in the population. Second, should another crisis occur, perhaps in the form of a new COVID-19 variant, our findings can guide a future response by suggesting where resources are best placed to maintain the health of the population. In addition, predicted mortality and morbidity rates from observed trends would allow the healthcare system to anticipate the impact of future pandemics. Finally, the first year of the pandemic held a lot of uncertainty, which caused a lot of stress and anxiety. Our results can be used to inform the public and healthcare providers what to expect in future pandemics.

Our study has number of strengths, including its inclusion of a large complete population, near-complete data during the first year of the pandemic on acute as well as ambulation care trends, monthly level analysis, open cohort study design, and the use of ARIMA modelling to project rates to account for any underlying trends and seasonality before the COVID-19 pandemic.

This study also has limitations. First, we were able to observe trends in outpatient visits and hospitalizations, but lacked detailed knowledge on what those health services were being used for. Second, while we can speculate on reasons for people not using health services, we lack knowledge of individual patient and healthcare provider motivations. Third, there are many other influences on the healthcare system we did not capture that could have influenced trends in ACSCs, such as visits for non-ACSCs such as COVID-19, delays and a backlog of surgery, which hospitals worked to correct, and health human resources issues with many providers experiencing illness and burnout. Next, with cross-national differences in COVID-19 pandemic-related shutdowns, public health and government mandates related to outbreak control, and resumptions to pre-pandemic levels of healthcare system functioning (70), these trends may have been different in different countries. Finally, it is still not clear what the persistent reductions in COPD, asthma and CHF signify, and it is possible they have led to poor outcomes beyond the first year of the pandemic. It is also not clear if the recovery of the other ACSC was sustained beyond that first year; thus, further study of the second years up until today is needed. Further studies are also needed to explore trends by the COVID-19 hotspot status.

## 5 Conclusion

In this province-based study, we demonstrated variation in outpatient visit trends for different ACSCs in the first year of the COVID-19 pandemic. No outpatient visit trends resulted in increased hospitalizations for any ACSC; however, reductions in rates of asthma, COPD, and CHF hospitalizations persisted. Future research should examine healthcare use beyond the first year of the pandemic to determine if these trends continued and/or how they reverberated to impact the health of the population.

## Data availability statement

The datasets presented in this article are not readily available because the dataset from this study is held securely in coded form at ICES. While legal data sharing agreements between ICES and data providers (e.g., healthcare organizations and government) prohibit ICES from making the dataset publicly available, access may be granted to those who meet pre-specified criteria for confidential access, available at www.ices.on.ca/DAS (email: das@ices.on.ca). The full dataset creation plan and underlying analytic code are available from the authors upon request, understanding that the computer programs may rely upon coding templates or macros that are unique to ICES and are, therefore, either inaccessible or may require modification. Requests to access the datasets should be directed to ICES data sharing, das@ices.on.ca.

## Ethics statement

The studies involving humans were approved by ICES. ICES is an independent non-profit institution and a prescribed entity under Personal Health Information Protection Act (PHIPA) in Ontario. Under section 45 of PHIPA, ICES is authorized to collect personal health information without consent to compile statistical information or analysis concerning managing, allocating resources to, monitoring, evaluating or planning for the health system. The studies were conducted in accordance with the local legislation and institutional requirements. Written informed consent for participation was not required from the participants or the participants' legal guardians/next of kin in accordance with the national legislation and institutional requirements.

## Author contributions

TK and AG were also involved in obtaining administrative data, analyzing data, and drafting the manuscript. MiP was involved in data analyses, visual data presentation and drafting the method section. DZ was involved in the literature search and drafting of the manuscript. TK, MiP, and AG had full access to all the data in the study and took responsibility for the integrity of the data and the accuracy of the data analysis, affirmed that the manuscript is an honest, accurate, and transparent account of the study being reported, no important aspects of the study have been omitted, any discrepancies from the study as planned have been explained. All authors were involved in study conception and design, interpretation of the data, critically revising the manuscript for accuracy and important intellectual content, and final approval of the version to be published, had full access to statistical reports and tables.

## Footnotes

^1^ https://datadictionary.ices.on.ca/Applications/DataDictionary/Default.aspx

## References

[1] Kendzerska T Zhu DT Gershon AS Edwards JD Peixoto C Robillard R. The effects of the health system response to the COVID-19 pandemic on chronic disease management: a narrative review. Risk Manag Healthc Policy. (2021) 14:575–84. doi: 10.2147/RMHP.S293471, PMID: 33623448 PMC7894869

[2] Sarmento J Rocha JVM Santana R. Defining ambulatory care sensitive conditions for adults in Portugal. BMC Health Serv Res. (2020) 20:754. doi: 10.1186/s12913-020-05620-9, PMID: 32799880 PMC7429814

[3] Kim AM Park JH Yoon TH Kim Y. Hospitalizations for ambulatory care sensitive conditions as an indicator of access to primary care and excess of bed supply. BMC Health Serv Res. (2019) 19:259. doi: 10.1186/s12913-019-4098-x, PMID: 31029134 PMC6487016

[4] Kwok ESH Clapham G Calder-Sprackman S. The impact of COVID-19 pandemic on emergency department visits at a Canadian academic tertiary care center. West J Emerg Med. (2021) 22:851–9. doi: 10.5811/westjem.2021.2.49626, PMID: 35353999 PMC8328159

[5] Wong LE Hawkins JE Langness S Murrell KL Iris P Sammann A. Where are all the patients? Addressing Covid-19 fear to encourage sick patients to seek emergency care. NEJM Catal Innov Care Deliv. (2020) 1:1–12. doi: 10.1056/CAT.20.0193

[6] Davies GA Alsallakh MA Sivakumaran S Vasileiou E Lyons RA Robertson C. Impact of COVID-19 lockdown on emergency asthma admissions and deaths: national interrupted time series analyses for Scotland and Wales. Thorax. (2021) 76:867–73. doi: 10.1136/thoraxjnl-2020-216380 33782079

[7] Babapoor-Farrokhran S Alzubi J Port Z Sooknanan N Ammari Z Al-Sarie M. Impact of COVID-19 on heart failure hospitalizations. SN Compr Clin Med. (2021) 3:2088–92. doi: 10.1007/s42399-021-01005-z, PMID: 34189405 PMC8225402

[8] He L Lu F Du X Long D Sang C Tang R. Impact of COVID-19 pandemic on hospital admissions of acute coronary syndrome: a Beijing inpatient database study. Lancet Reg Health West Pac. (2022) 19:100335. doi: 10.1016/j.lanwpc.2021.100335 34927111 PMC8665660

[9] Leung WCY Lau EHY Kwan P Chang RS. Impact of COVID-19 on seizure-related emergency attendances and hospital admissions – a territory-wide observational study. Epilepsy Behav. (2021) 115:107497. doi: 10.1016/j.yebeh.2020.107497, PMID: 33317939 PMC7505596

[10] Mayoral E Rave R Rodriguez de Vera P Rojo-Martinez G Olveira G Aguilar-Diosdado M. Temporal trends in hospitalizations due to diabetes complications during COVID-19 pandemic in Andalusia, Spain. BMJ Open Diabetes Res Care. (2022) 10:e002623. doi: 10.1136/bmjdrc-2021-002623, PMID: 35351686 PMC8965863

[11] Ahrens SM Ostendorf AP Lado FA Arnold ST Bai S Bensalem-Owen MK. Impact of the COVID-19 pandemic on epilepsy center practice in the United States. Neurology. (2022) 98:e1893–901. doi: 10.1212/WNL.0000000000200285, PMID: 35292559 PMC9141627

[12] Kendzerska T Zhu DT Pugliese M Manuel D Sadatsafavi M Povitz M. Trends in all-cause mortality and inpatient and outpatient visits for ambulatory care sensitive conditions during the first year of the COVID-19 pandemic: a population-based study. J Hosp Med. (2022) 17:726–37. doi: 10.1002/jhm.12920 35929531 PMC9539068

[13] Lawless M Burgess M Bourke S. Impact of COVID-19 on hospital admissions for COPD exacerbation: lessons for future care. Medicina (Kaunas). (2022) 58:66. doi: 10.3390/medicina58010066 35056374 PMC8778793

[14] De Filippo O D'Ascenzo F Angelini F Bocchino PP Conrotto F Saglietto A. Reduced rate of hospital admissions for ACS during Covid-19 outbreak in northern Italy. N Engl J Med. (2020) 383:88–9. doi: 10.1056/NEJMc2009166, PMID: 32343497 PMC7224608

[15] Kenyon CC Hill DA Henrickson SE Bryant-Stephens TC Zorc JJ. Initial effects of the COVID-19 pandemic on pediatric asthma emergency department utilization. J Allergy Clin Immunol Pract. (2020) 8:2774–2776.e1. doi: 10.1016/j.jaip.2020.05.045, PMID: 32522565 PMC7483361

[16] Stohr E Aksoy A Campbell M Al Zaidi M Ozturk C Vorloeper J. Hospital admissions during Covid-19 lock-down in Germany: differences in discretionary and unavoidable cardiovascular events. PLoS One. (2020) 15:e0242653. doi: 10.1371/journal.pone.0242653, PMID: 33216804 PMC7678984

[17] Rennert-May E Leal J Thanh NX Lang E Dowling S Manns B. The impact of COVID-19 on hospital admissions and emergency department visits: a population-based study. PLoS One. (2021) 16:e0252441. doi: 10.1371/journal.pone.0252441, PMID: 34061888 PMC8168854

[18] Wongtanasarasin W Srisawang T Yothiya W Phinyo P. Impact of national lockdown towards emergency department visits and admission rates during the COVID-19 pandemic in Thailand: a hospital-based study. Emerg Med Australas. (2021) 33:316–23. doi: 10.1111/1742-6723.13666, PMID: 33070468

[19] White F. Application of disease etiology and natural history to prevention in primary health care: a discourse. Med Princ Pract. (2020) 29:501–13. doi: 10.1159/000508718, PMID: 32422632 PMC7768156

[20] Statistics Canada. (2021) Table 17-10-0009-01 Population estimates, quarterly. Release date: 2021-09-29. Available at: https://www150.statcan.gc.ca/t1/tbl1/en/tv.action?pid=1710000901.

[21] ICES Investigative Report. Improving health care data in Ontario. Toronto: Institute for Clinical Evaluative Sciences (2005).

[22] Juurlink DPC Croxford R Chong A Austin P Tu J Laupacis A. Canadian Institute for Health Information Discharge Abstract Database: A validation study. Toronto: Institute for Clinical Evaluative Sciences (2006).

[23] Goel V. Patterns of health care in Ontario. 2nd ed. Ottawa: Canadian Medical Association [for] the Institute for Clinical Evaluative Sciences in Ontario (1996) 354 pp.

[24] Canadian Instititute for Health Information (2021) Physician billing codes in response to COVID-19. Available at: https://www.cihi.ca/en/physician-billing-codes-in-response-to-covid-19#ON.

[25] Rodrigues G. Ontario government declares state of emergency amid coronavirus pandemic. Global News (2020).

[26] Cameron-Blake E Breton C Sim P Tatlow H Hale T Wood A. Variation in the Canadian provincial and territorial responses to COVID-19 (2021).

[27] Fasken (2020). Canadian Health Sector: Updates Related to COVID-19 in March 2020. Available at: https://www.fasken.com/en/knowledge/2020/03/30-covid19-canadian-health-sector-updates-march-2020.

[28] Government of Canada. COVID-19 epidemiology update: Current sitation. Available at: https://health-infobase.canada.ca/covid-19/current-situation.html.

[29] Text Table A. International Statistical Classification of Diseases and Related Health Problems, 10th Revision, Canada (ICD-10-CA) codes used to define ambulatory care sensitive conditions Statistics Canada. Date modified: 2017-12-20. Available at: https://www150.statcan.gc.ca/n1/pub/82-003-x/2017012/article/54891/tbl/ttbla-eng.htm.

[30] Canadian Institute for Health Information. Ambulatory Care Sensitive Conditions. Available at: www.cihi.ca/en/indicators/ambulatory-care-sensitive-conditions.

[31] Box GEP Jenkins GM Reinsel GC. Time series analysis: forecasting and control. Hoboken, NJ: John Wiley (2008).

[32] Census.gov. Time series research staff, Center for Statistical Research and Methodology, U.S. Census Bureau Reference Manual for X-13ARIMA-SEATS 2017. Available at: http://www.census.gov/srd/www/x13as/.

[33] Dagum EB. The X11ARIMA/88 seasonal adjustment method – foundations and User's manual. Time Series Research and Analysis Division (1988).

[34] Gomez V Maravall A. Automatic modeling methods for univariate series In: Pena D Tiao GC Tsay RS, editors. A course in time series analysis. New York, NY: John Wiley and Sons (2001)

[35] Schaffer AL Dobbins TA Pearson SA. Interrupted time series analysis using autoregressive integrated moving average (ARIMA) models: a guide for evaluating large-scale health interventions. BMC Med Res Methodol. (2021) 21:58. doi: 10.1186/s12874-021-01235-8, PMID: 33752604 PMC7986567

[36] Hyndman R Kostenko AV. Minimum sample size requirements for seasonal forecasting models. Foresight Int J Appl Forecast. (2007) 6:12–5.

[37] Huang YT Lee YC Hsiao CJ. Hospitalization for ambulatory-care-sensitive conditions in Taiwan following the SARS outbreak: a population-based interrupted time series study. J Formos Med Assoc. (2009) 108:386–94. doi: 10.1016/S0929-6646(09)60082-6, PMID: 19443292 PMC7135451

[38] Czeisler MÉ Marynak K Clarke KE Salah Z Shakya I Thierry JM. Delay or avoidance of medical care because of COVID-19–related concerns — United States, June 2020. MMWR Morb Mortal Wkly Rep. (2020) 69:1250–7. doi: 10.15585/mmwr.mm6936a4, PMID: 32915166 PMC7499838

[39] Nab M van Vehmendahl R Somers I Schoon Y Hesselink G. Delayed emergency healthcare seeking behaviour by Dutch emergency department visitors during the first COVID-19 wave: a mixed methods retrospective observational study. BMC Emerg Med. (2021) 21:56. doi: 10.1186/s12873-021-00449-9, PMID: 33932988 PMC8087882

[40] Guimaraes RA Policena GM Paula H Pedroso CF Pinheiro RS Itria A. Analysis of the impact of coronavirus disease 19 on hospitalization rates for chronic non-communicable diseases in Brazil. PLoS One. (2022) 17:e0265458. doi: 10.1371/journal.pone.0265458 35324951 PMC8947087

[41] Banerjee A Chen S Pasea L Lai AG Katsoulis M Denaxas S. Excess deaths in people with cardiovascular diseases during the COVID-19 pandemic. Eur J Prev Cardiol. (2021) 28:1599–609. doi: 10.1093/eurjpc/zwaa155, PMID: 33611594 PMC7928969

[42] Santi L Golinelli D Tampieri A Farina G Greco M Rosa S. Non-COVID-19 patients in times of pandemic: emergency department visits, hospitalizations and cause-specific mortality in northern Italy. PLoS One. (2021) 16:e0248995. doi: 10.1371/journal.pone.0248995, PMID: 33750990 PMC7984614

[43] Haki C Deniz O. The impact of home quarantine during COVID-19 lockdown on neurological hospitalizations, in-hospital mortality, and acute ischemic stroke management in older patients without COVID-19. Clin Neurol Neurosurg. (2022) 212:107027. doi: 10.1016/j.clineuro.2021.107027, PMID: 34839154 PMC8604567

[44] Hangartner N Di Gangi S Elbl C Senn O Bisatz F Fehr T. Impact of the COVID-19 pandemic on emergency outpatient consultations and admissions of non-COVID-19 patients (ECCO)-a cross-sectional study. PLoS One. (2022) 17:e0269724. doi: 10.1371/journal.pone.0269724, PMID: 35687575 PMC9187104

[45] Poucineau J Delory T Lapidus N Hejblum G Chouaid C Le Coeur S. Hospital admissions and mortality for acute exacerbations of COPD during the COVID-19 pandemic: a nationwide study in France. Front Med (Lausanne). (2022) 9:995016. doi: 10.3389/fmed.2022.995016, PMID: 36186789 PMC9522972

[46] Pujolar G Oliver-Angles A Vargas I Vazquez ML. Changes in access to health services during the COVID-19 pandemic: a scoping review. Int J Environ Res Public Health. (2022) 19:1749. doi: 10.3390/ijerph19031749, PMID: 35162772 PMC8834942

[47] Kaufman HW Chen Z Niles J Fesko Y. Changes in the number of US patients with newly identified Cancer before and during the coronavirus disease 2019 (COVID-19) pandemic. JAMA Netw Open. (2020) 3:e2017267. doi: 10.1001/jamanetworkopen.2020.17267, PMID: 32749465 PMC7403918

[48] Olsen SJ Azziz-Baumgartner E Budd AP Brammer L Sullivan S Pineda RF. Decreased influenza activity during the COVID-19 pandemic-United States, Australia, Chile, and South Africa, 2020. Am J Transplant. (2020) 20:3681–5. doi: 10.1111/ajt.16381, PMID: 33264506 PMC7753605

[49] Chow EJ Uyeki TM Chu HY. The effects of the COVID-19 pandemic on community respiratory virus activity. Nat Rev Microbiol. (2022) 25:195–210. doi: 10.1038/s41579-022-00807-9 PMC9574826 36253478

[50] Datta BK Ansa BE George V. An analytical model of population level chronic conditions and COVID-19 related hospitalization in the United States. BMC Public Health. (2022) 22:208. doi: 10.1186/s12889-022-12531-3 35101029 PMC8803409

[51] Kaye L Theye B Smeenk I Gondalia R Barrett MA Stempel DA. Changes in medication adherence among patients with asthma and COPD during the COVID-19 pandemic. J Allergy Clin Immunol Pract. (2020) 8:2384–5. doi: 10.1016/j.jaip.2020.04.053, PMID: 32371047 PMC7194036

[52] Skene IP Pfeffer PE. Improved asthma control during the COVID-19 pandemic: are there lessons to be learnt? Thorax. (2021) 76:852–3. doi: 10.1136/thoraxjnl-2021-216930, PMID: 33837141

[53] Moore WC Ledford DK Carstens DD Ambrose CS. Impact of the COVID-19 pandemic on incidence of asthma exacerbations and hospitalizations in US subspecialist-treated patients with severe asthma: results from the CHRONICLE study. J Asthma Allergy. (2022) 15:1195–203. doi: 10.2147/JAA.S363217, PMID: 36068863 PMC9441176

[54] Birkmeyer JD Barnato A Birkmeyer N Bessler R Skinner J. The impact of the COVID-19 pandemic on hospital admissions in the United States. Health Aff (Millwood). (2020) 39:2010–7. doi: 10.1377/hlthaff.2020.00980, PMID: 32970495 PMC7769002

[55] Dayal D Gupta S Raithatha D Jayashree M. Missing during COVID-19 lockdown: children with onset of type 1 diabetes. Acta Paediatr. (2020) 109:2144–6. doi: 10.1111/apa.15443, PMID: 32575149

[56] Gujral UP Johnson L Nielsen J Vellanki P Haw JS Davis GM. Preparedness cycle to address transitions in diabetes care during the COVID-19 pandemic and future outbreaks. BMJ Open Diabetes Res Care. (2020) 8:e001520. doi: 10.1136/bmjdrc-2020-001520, PMID: 32690631 PMC7385737

[57] Shah SA Quint JK Nwaru BI Sheikh A. Impact of COVID-19 national lockdown on asthma exacerbations: interrupted time-series analysis of English primary care data. Thorax. (2021) 76:860–6. doi: 10.1136/thoraxjnl-2020-216512, PMID: 33782080 PMC8011425

[58] Shah SA Quint JK Sheikh A. Impact of COVID-19 pandemic on asthma exacerbations: retrospective cohort study of over 500,000 patients in a national English primary care database. Lancet Reg Health Eur. (2022) 19:100428. doi: 10.1016/j.lanepe.2022.100428, PMID: 35756853 PMC9213032

[59] Papafaklis MI Katsouras CS Tsigkas G Toutouzas K Davlouros P Hahalis GN. "Missing" acute coronary syndrome hospitalizations during the COVID-19 era in Greece: medical care avoidance combined with a true reduction in incidence? Clin Cardiol. (2020) 43:1142–9. doi: 10.1002/clc.23424, PMID: 32691901 PMC7404667

[60] Arabi A Ahmad F Al-Suwaidi J Al-Qahtani A Asaad N Rafie I. The impact of COVID-19 outbreak on cardiovascular admissions. Heart Views. (2020) 21:153–6. doi: 10.4103/HEARTVIEWS.HEARTVIEWS_141_20, PMID: 33688405 PMC7899007

[61] Schwarz V Mahfoud F Lauder L Reith W Behnke S Smola S. Decline of emergency admissions for cardiovascular and cerebrovascular events after the outbreak of COVID-19. Clin Res Cardiol. (2020) 109:1500–6. doi: 10.1007/s00392-020-01688-9, PMID: 32749557 PMC7399595

[62] Severino P D'Amato A Saglietto A D'Ascenzo F Marini C Schiavone M. Reduction in heart failure hospitalization rate during coronavirus disease 19 pandemic outbreak. ESC Heart Fail. (2020) 7:4182–8. doi: 10.1002/ehf2.13043 33094929 PMC7754919

[63] Frankfurter C Buchan TA Kobulnik J Lee DS Luk A McDonald M. Reduced rate of hospital presentations for heart failure during the COVID-19 pandemic in Toronto, Canada. Can J Cardiol. (2020) 36:1680–4. doi: 10.1016/j.cjca.2020.07.006, PMID: 32682855 PMC7366087

[64] Simoneau T Greco KF Hammond A Nelson K Gaffin JM. Impact of the COVID-19 pandemic on pediatric emergency department use for asthma. Ann Am Thorac Soc. (2021) 18:717–9. doi: 10.1513/AnnalsATS.202007-765RL, PMID: 33272107 PMC8009002

[65] Sevinc C Tertemiz KC Atik M Guler N Ulusoy M Coskun F. How were non-COVID pulmonary patients and diseases affected from COVID-19 pandemic period? Turk Thorac J. (2021) 22:149–53. doi: 10.5152/TurkThoracJ.2021.20249, PMID: 33871339 PMC8051290

[66] Bamaga AK Alharbi O Bajuaifer M Batarfi A Althobaiti KH AlQusaibi B. The effect of the COVID-19 pandemic on emergency department visits for neurological diseases in Saudi Arabia. Cureus. (2020) 12:e12200. doi: 10.7759/cureus.12200, PMID: 33489609 PMC7816827

[67] Davico C Marcotulli D Lux C Calderoni D Terrinoni A Di Santo F. Where have the children with epilepsy gone? An observational study of seizure-related accesses to emergency department at the time of COVID-19. Seizure. (2020) 83:38–40. doi: 10.1016/j.seizure.2020.09.025, PMID: 33080483 PMC7534601

[68] Mostacci B Licchetta L Cacciavillani C Di Vito L Ferri L Menghi V. The impact of the COVID-19 pandemic on people with epilepsy. An Italian survey and a global perspective. Front Neurol. (2020) 11:613719. doi: 10.3389/fneur.2020.613719, PMID: 33391172 PMC7775598

[69] Adan GH Mitchell JW Marson T. Epilepsy care in the COVID-19 era. Clin Med (Lond). (2020) 20:e104–6. doi: 10.7861/clinmed.2020-0207, PMID: 32518102 PMC7385807

[70] Cassell K Zipfel CM Bansal S Weinberger DM. Trends in non-COVID-19 hospitalizations prior to and during the COVID-19 pandemic period, United States, 2017-2021. Nat Commun. (2022) 13:5930. doi: 10.1038/s41467-022-33686-y, PMID: 36209210 PMC9546751

