# Supplementary material for: Trends in outpatient and inpatient visits for separate ambulatory-care-sensitive conditions during the first year of the COVID-19 pandemic: a province-based study
Source: Front Public Health. 2023 Dec 18;11:1251020. doi: 10.3389/fpubh.2023.1251020 (PMC10759216; doi:10.3389/fpubh.2023.1251020)
Supplement: Supplementary file 1 [file Table_1.DOCX]

**Title**: Trends in Outpatient and Inpatient Visits for Separate Ambulatory-Care-Sensitive Conditions during the First Year of the COVID-19 Pandemic: A Province-Based Study

**Authors**: Tetyana Kendzerska, David T. Zhu, Michael Pugliese, Douglas Manuel, Mohsen Sadatsafavi, Marcus Povitz, Therese A Stukel, Teresa To, Shawn D. Aaron, Sunita Mulpuru, Melanie Chin, Claire E. Kendall, Kednapa Thavorn, Rebecca Robillard, Andrea S. Gershon

**Text S1:** Details on the ARIMA procedure.

**Tables:**

**Table S1.** Details on ICES databases used.

**Table S2.** Definitions of outpatient and inpatient visits for seven chronic ambulatory care sensitive conditions (ACSCs) from health administrative data.

**Table S3**. Observed and projected monthly rates and 95% confidence intervals (CI) in subgroups estimated by ARIMA Models for outpatient and inpatient (emergency department visits and hospitalizations) care visits for **COPD**: rates were calculated as the number of events per 100,000 people at risk.

**Table S4**. Observed and projected monthly rates and 95% confidence intervals (CI) in subgroups estimated by ARIMA Models for outpatient and inpatient (emergency department visits and hospitalizations) care visits for **asthma**: rates were calculated as the number of events per 100,000 people at risk.

**Table S5**. Observed and projected monthly rates and 95% confidence intervals (CI) in subgroups estimated by ARIMA Models for outpatient and inpatient (emergency department visits and hospitalizations) care visits for **angina**: rates were calculated as the number of events per 100,000 people at risk.

**Table S6**. Observed and projected monthly rates and 95% confidence intervals (CI) in subgroups estimated by ARIMA Models for outpatient and inpatient (emergency department visits and hospitalizations) care visits for **congestive heart failure** **(CHF)**: rates were calculated as the number of events per 100,000 people at risk.

**Table S7**. Observed and projected monthly rates and 95% confidence intervals (CI) in subgroups estimated by ARIMA Models for outpatient and inpatient (emergency department visits and hospitalizations) care visits for **diabetes**: rates were calculated as the number of events per 100,000 people at risk.

**Table S8**. Observed and projected monthly rates and 95% confidence intervals (CI) in subgroups estimated by ARIMA Models for outpatient and inpatient (emergency department visits and hospitalizations) care visits for **hypertension**: rates were calculated as the number of events per 100,000 people at risk.

**Table S9**. Observed and projected monthly rates and 95% confidence intervals (CI) in subgroups estimated by ARIMA Models for outpatient and inpatient (emergency department visits and hospitalizations) care visits for **epilepsy**: rates were calculated as the number of events per 100,000 people at risk.

**Figures**:

**Figure S1**: (A) A flow chart for “manual” ARIMA model selection method that is typically used (Box-Jenkins method); (B) A flow chart for the X13 automated model selection procedure used in our study.

**References**

**Text S1:** Details on the ARIMA procedure.

We identified healthcare usage using CIHI’s Discharge Abstract Database (DAD) for inpatient acute care, CIHI’s National Ambulatory Care Reporting System (NACRS) for emergency department visits, and OHIP for outpatient visits. We captured deaths using the Registered Persons Database. Monthly rates are calculated as the number of events per 100,000 person-days. A person accrued person-days in a month until they either died or lost their OHIP eligibility.

We used autoregressive integrated moving average (ARIMA) models to calculate projected rates. ARIMA models regress an outcome on its past values, fitting autoregressive (AR) and moving-average (MA) terms to a time series once it has been made stationary, often through differencing (integration). Seasonal terms can be added to the model to account for the correlation structure of regular seasonal patterns in a time series. These models are sometimes referred to as seasonal ARIMA models (SARIMA).

AR and MA terms are typically selected manually using the method described by Box and Jenkins [1]. However, the volume of projections required for this study makes manual ARIMA model selection impractical. Instead, we used SAS software’s adaption of the United States Census Bureau’s X-13ARIMA-SEATS program [2] (SAS software, version 9.4). The X13 procedure includes programming for seasonal adjustments using the X-11-ARIMA procedure [3], and automated model selection using TRAMO (time series regression with ARIMA noise, missing values, and outliers) [4].

The automated model selection procedure is complex, a full description can be found in the US census bureau’s reference manual [2]. Briefly, a default model is estimated along with residual diagnostics to be compared with the selected model at a later step. Empirical root tests determine the orders of differencing to make the series stationary and an iterative process is used to fit multiple models with different AR and MA terms. The best model is selected using BIC and then compared to the default model. The better performing model is retained, and a final model evaluation is performed during which orders of differencing or model terms may be adjusted.

We used the final model for each outcome to create projected monthly rates for 13 months following February 2020. We compared observed monthly rates with projected rates, considering observed rates outside of the projected 95% confidence intervals to be significantly different [5]. Comparison between projected and observed rates are presently graphically as a time series and in tabular form using mean rates across four time periods. We performed all data analyses in SAS software version 9.4 using SAS Enterprise guide version 7.15.3.

**Table S1.** Details on ICES databases used.

**General comments**: Since 1991, ICES (www.ices.on.ca) has housed high-quality administrative datasets on publicly funded services provided, including individual-level information on demographics, physician claims, procedures, hospitalization, and emergency visits within Ontario.[6, 7] Using health administrative databases limits information biases such as recall bias, observation bias, and reporting bias. The accuracy of these datasets has been previously validated. [8, 9] A unique provincial personal health number helps to ensure reliable linkages between databases.[10] A description of the ICES datasets is available at https://datadictionary.ices.on.ca/Applications/DataDictionary/Default.aspx. However, health administrative databases in Ontario initially were created for administration purposes, thus, are prompt to misclassification bias, less detailed in their clinical contents, and provide a limited ability to control confounding.[7, 10-12]

| **Database** | **Available from** | **Description** | **Update frequency** | **Specific database-related limitations** |
| --- | --- | --- | --- | --- |
| **Registered Persons Database (RPDB)** | 1991 | RPDB includes information on every individual who has been ever issued an Ontario Health Insurance Plan (OHIP) card. RPDB captures almost all of Ontario’s 13.4 million residents. Raw data updates are provided to ICES by the Ontario Ministry of Health and Long-Term Care (MOHLTC) under a specific data sharing agreement. The RPDB file contains the individual health card number, as well as demographics and personally identifiable information (e.g., surname, given names, sex, date of birth, the earliest date of coverage, last date of contact with the health care system and residential postal code). The RPDB forms the spine for ICES record linkage. Using a highly confidential and secure proprietary algorithm, each OHIP number in RPDB and any other health data with an OHIP number is uniquely converted to an anonymous ICES Identifier. | Monthly | Individuals without OHIP are not captured; however, Ontario has universal health coverage. |
| **Discharge Abstract Database (DAD)*** | 1988 | Submission of information to the DAD is mandatory in Ontario. The Canadian Institute for Health Information (CIHI) periodically re-abstracts data from charts to assess the quality of DAD. The high validity of the DAD is related to many factors, including the extensive training of coders in colleges, rigourous and comprehensive coding guidelines, and the case mix-based hospital funding model.  This database includes data on up to 16 diagnoses and procedures based on ICD-9 codes (up to March 2002) and 25 diagnoses and procedures based on ICD-10-CA (April 1, 2002, and onward) performed for each hospital admission, including ICU.  All diagnosis codes listed on the DAD abstract are classified according to type. Multiple types of diagnoses are recorded in the DAD abstract, including most responsible diagnosis, preadmit comorbidity, postadmit comorbidity, secondary diagnoses, and admitting diagnosis. | Quarterly | # of digits used were three for ICD-9 (up to March 31, 2002) and five for ICD-10-CA (April 1, 2002, and onward).  All hospital diagnoses are based on exact codes; thus, they can be affected by misclassification bias due to errors in coding. |
| **National Ambulatory Care Reporting System (NACRS)** | ED: 2002 | This database includes data on up to 10 diagnoses and procedures for emergency room (ED) and urgent care visits. The Canadian Emergency Department Diagnoses Shortlist includes more than 800 diagnoses in common terms, which are mapped to ICD-10-CA codes. The Emergency Department Intervention Value Set includes a list of 173 clinical (common) terms for interventions that are most common/relevant to ED encounters. The terms are mapped to Canadian Classification of Health Interventions (CCI) codes. | Quarterly | All diagnoses and procedures are based on exact codes; thus, they can be affected by misclassification bias due to errors in coding. |
| **OHIP Claims Database** | 1991 | The OHIP is the universal health insurance system that provides almost all Ontario residents with health care services free at the point of delivery based on an OHIP card and its unique 10-digit identifier.[7]  The data cover all healthcare providers who can claim  under OHIP (this includes physicians, groups, laboratories, and out-of-province providers). Approximately 95% of specialists and 50% of primary care physicians receive most of their income through fee-for-service (FFS) billings to OHIP. To ensure that OHIP data accurately reflect the utilization of physician services in Ontario, all physicians (except the few hundred family physicians working in Community Health Centres) must submit shadow billings for their non-FFS services. Physicians are often provided with cash incentives to encourage them to shadow bills.  This database includes data on all physician billing and technical fees for procedures such as polysomnography. | Monthly | All physician claims diagnoses are based on three-digit modified ICD-8 codes.  Some health services are not universal and are paid for by OHIP based on eligibility criteria, such as prescription drugs which are publicly funded for those under the age of 25, those over the age of 64, those living in a long-term care home or receiving social assistance. |
| **COVID-19-specific datasets#** |  |  |  |  |
| COVID19 Integrated Testing Data (C19INTGR) | Jan 2020 | ICES-derived comprehensive dataset of all available COVID-19 diagnostic laboratory results in Ontario.[13]  The C19INTGR is derived from 3 data sources: 1) Ontario Laboratories Information System (OLIS) contains COVID-19 testing episodes using standard PCR tests from January 2020 to current; 2) Distributed testing data from laboratories within the COVID-19 Diagnostic Network, with results only up to April 13, 2020 (prior to a requirement to report all test results in OLIS, the results of tests performed by laboratories that were part of this network were compiled by PHO in a separate database); and 3) Public Health Case & Contact Management (CCM) Solution, formerly known as the integrated Public Health Information System (iPHIS), a client-level dataset (not testing episodes) for individuals who are confirmed positive for COVID-19 based on the provincial case definition, from January 2020 to current. | Monthly |  |
| OLIS COVID-19 Laboratory Data (OLISC19) | Jan 2020 | OLIS provides lab results of patients from all Public  Health Ontario laboratories and a number of hospitals  and community laboratories.[14]  Variables included in this dataset include province, postal code, date of birth, sex, encrypted health card number, specimen collection date, result release date, whether a COVID test was done, COVID-19 test result (Positive/Presumptive > Indeterminate > Negative > Pending > Cancelled > Rejected). | Monthly | The number of individuals who tested positive in OLIS is ~90% of the cases reported by the Ministry of Health, which uses the iPHIS.  Earlier in the pandemic, not all laboratories contributed their lab results to the OLIS, resulting in under-reporting COVID-19 tests and positive COVID-19 cases.  Due to the time required for the transportation and processing of specimens, it takes up to six days for approximately 95% of the results to be finalized and reported for a given testing date. |
| Case and Contact Management System (CCM) | Jan 2020 | The Case and Contact Management System (CCM) is a central data repository for COVID-19 case and contact management and reporting in Ontario.[15] This information is used for local, provincial and national surveillance.  Between July and August 2020, CCM replaced the iPHIS for COVID-19 for most health units in the province. COVID-19 cases in iPHIS were migrated over to CCM with key reporting elements. iPHIS is used for collecting information on all other reportable diseases in Ontario. | Weekly | The number of cases of COVID-19 in CCM is an underestimate of the actual numbers since not all people with COVID-19 develop symptoms, seek medical treatment or testing, and therefore, the disease goes unreported. |

* The Discharge Abstract Database (DAD) is a national database established by the Canadian Institute for Health Information (CIHI) to compile data from acute care institutions across the country

#COVID-19-specific datasets: Initially, only a few laboratories in Ontario were performing SARS-CoV-2 testing, and test results were not stored in a centralized repository. Eventually, testing became more widespread, and results were centralized, and on **April 7, 2020**, ICES started receiving a daily feed of SARS-CoV-2 real-time polymerase chain reaction test results contained in the Ontario Laboratories Information System (OLIS). ICES collaborated closely with teams at the Ministry of Health, Public Health Ontario, and clinical subject matter experts to interpret, validate, and develop an algorithm to transform these data into a research-friendly format. The code to parse relevant test results from these data was published under an open-source license and was subsequently used by the Ministry of Health and Ontario Health for their COVID-19 analytics. Adoption of this code by other organizations that were receiving the same OLIS data feed enabled consistent interpretation of SARS-CoV-2 test results and facilitated uniform reporting of COVID-19 metrics. Information on COVID-19 cases, such as symptoms, epidemiological contacts and risk factors, are not complete in the OLIS data nor are captured in other health administrative databases. Thus, to enable more descriptive reporting on COVID-19 cases, ICES started receiving daily feeds of the Public Health Case and Contact Management (CCM) Solutions database. CCM was used in combination with OLIS to identify all individuals confirmed positive for SARS-CoV-2 because there were differences in capture rates between the two data sources: OLIS captures approximately ~90% of all confirmed cases that are reported in CCM, but approximately ~6% of cases in CCM are not linkable to other ICES data holdings.

CCI, Canadian Classification of Health Interventions; CIHI, Canadian Institute for Health Information; CCM, Case and Contact Management System; DAD, Discharge Abstract Database; FFS, fee-for-service; iPHIS, Integrated Public Health Information System; OHIP, Ontario Health Insurance Plan; OLIS, Ontario Laboratories Information System; RPDB, Registered Persons Database.

**Table S2.** Definitions of outpatient and inpatient visits for seven chronic ambulatory care sensitive conditions (ACSCs) from health administrative data*: angina, asthma, CHF, COPD, diabetes, epilepsy, and hypertension.

| **Ambulatory care sensitive condition** | **ICD 10 CA code range** | **OHIP diagnostic codes (to define outpatient visits)** |
| --- | --- | --- |
| **Diabetes** | E10.0, E10.1, E10.63, E10.64, E10.9, E11.0, E11.1, E11.63, E11.64, E11.9, E13.0, E13.1, E13.63, E13.64, E13.9, E14.0, E14.1, E14.63, E14.64, E14.9 | 250 |
| **Chronic obstructive pulmonary disease (COPD)** | J41, J42, J43, J44, or J47  Or  J100, J110, J12-J16, J18, J20, J21, J22 only if a diagnosis code of J44 (any dxtype) is also present in the discharge record. | 491, 492, 496 |
| **Asthma** | J45 | 493 |
| **Angina** | I20, I23.82, I24.0, I24.8, or I24.9, **excluding cases with cardiac procedures** (see below) that are not coded as abandoned after onset | 413 |
| **Grand mal status and other epileptic convulsions** | G40 or G41 | 345 |
| **Congestive heart failure (CHF) and pulmonary edema** | I50 or J81, **excluding cases with cardiac procedures** (see below) that are not coded as abandoned after onset | 428 |
| **Hypertension** | I10.0, I10.1, or I11, **excluding cases with cardiac procedures** (see below) that are not coded as abandoned after onset | 401, 402 |
| **Exclusion qualifiers** | **one of these procedure or intervention codes are not ACSC admissions** |  |
| **Surgical intervention exclusions** | From hospitalizations with a MRDx of CHF, hypertension, or angina (see above), excluding any hospitalizations where any cardiac procedures (CCI code) for the discharge that meet the following criteria:  Exclude CCI codes beginning with 1HA58, 1HA80, 1HA87, 1HB53, 1HB54, 1HB55, 1HB87, 1HD53, 1HD54, 1HD55, 1HH59, 1HH71, 1HJ76, 1HJ82, 1HM57, 1HM78, 1HM80, 1HN71, 1HN80, 1HN87, 1HP76, 1HP78, 1HP80, 1HP82, 1HP83, 1HP87, 1HR71, 1HR80, 1HR84, 1HR87, 1HS80, 1HS90, 1HT80, 1HT89, 1HT90, 1HU80, 1HU90, 1HV80, 1HV90, 1HW78, 1HW79, 1HX71, 1HX78, 1HX79, 1HX80, 1HX83, 1HX86, 1HX87, 1HY85, 1HZ53, 1HZ54, 1HZ55, 1HZ56, 1HZ57, 1HZ59, 1HZ80, 1HZ85, 1HZ87, 1IF83, 1IJ50, 1IJ55, 1IJ57, 1IJ76, 1IJ80, 1IK57, 1IK80, 1IK87, 1IN84, 1LA84, 1LC84, 1LD84, 1IJ86  AND not equal to (1HZ53LAKP, 1HZ55LAKP)  AND not equal to abandoned at onset |  |

ICD-10 is the 10th revision of the International Statistical Classification of Diseases and Related Health Problems (ICD); MRDx, most responsible diagnosis; OHIP, the Ontario Health Insurance Plan.

*Adapted from the Hospitalizations for Ambulatory Care Sensitive Conditions (ACSC) Resource for Indicator Standards (RIS); Health Analytics Branch, Ministry of Health and Long-Term Care [16]. The most responsible diagnosis (MRDx) is the diagnosis that contributes to the greatest extent to the length of stay in a hospital, and may or may not be the original reason for admission.

**Table S3**. Observed and projected monthly rates and 95% confidence intervals (CI) in subgroups estimated by ARIMA Models for outpatient and inpatient (emergency department visits and hospitalizations) care visits for **COPD**: rates were calculated as the number of events per 100,000 people at risk. Similar periods in previous years (2016-2019) were used to calculate projected rates.

| Population  Rates | Observed | Projected  (95% CI) | Observed | Projected  (95% CI) | Observed | Projected  (95% CI) | Observed | Projected  (95% CI) |
| --- | --- | --- | --- | --- | --- | --- | --- | --- |
|  | **Pre-COVID**  Jan-Feb 2020 | | **Wave I**  Mar-May 2020 | | **Summer Lull**  Jun-Aug 2020 | | **Wave II** Sep 2020 - Mar 2021 | |
| ***Age***, years | | | | | | | | |
| **35-49 years old** |  |  |  |  |  |  |  |  |
| Primary care | 29.67 | 27.33 (23.90-31.25) | 25.25 | 24.81 (21.68-28.39) | 20.36 | 22.14 (19.33-25.37) | **21.39** | 25.64 (22.23-29.59) |
| Specialty care | 12.80 | 12.44 (10.57-14.63) | **10.40** | 12.89 (10.79-15.40) | 10.48 | 11.47 (9.23-14.24) | 12.94 | 12.22 (9.31-16.05) |
| Virtual visit | 0.76 | 1.05 (0.65-1.46) | **23.91** | 0.78 (0.32-1.24) | **20.71** | 0.78 (0.18-1.38) | **22.94** | 0.78 (0.00-1.56) |
| ED visits | 6.16 | 6.10 (5.06-7.35) | **3.79** | 5.20 (4.23-6.38) | **2.12** | 4.18 (3.37-5.18) | **2.47** | 5.76 (4.63-7.17) |
| Hospitalizations | 1.34 | 1.41 (0.98-2.03) | 0.95 | 1.19 (0.83-1.72) | **0.43** | 0.86 (0.60-1.24) | **0.62** | 1.30 (0.90-1.89) |
| **50-64 years old** |  |  |  |  |  |  |  |  |
| Primary care | 154.40 | 150.06 (135.15-166.60) | 137.45 | 147.05 (132.17-163.62) | 110.77 | 122.69 (109.96-136.90) | **116.20** | 142.44 (126.98-159.81) |
| Specialty care | 87.26 | 82.71 (74.75-91.50) | **76.29** | 92.62 (83.41-102.86) | 76.22 | 81.29 (72.74-90.84) | 88.58 | 85.67 (76.24-96.30) |
| Virtual visit | 4.22 | 4.20 (3.38-5.02) | **146.83** | 4.70 (3.82-5.58) | **134.00** | 4.57 (3.51-5.63) | **138.46** | 5.02 (3.70-6.34) |
| ED visits | 44.77 | 47.37 (39.76-56.45) | **25.82** | 42.52 (35.69-50.67) | **21.51** | 34.71 (29.13-41.36) | **19.15** | 43.77 (36.62-52.32) |
| Hospitalizations | 17.31 | 17.29 (14.40-20.77) | **9.92** | 15.48 (12.89-18.60) | **8.05** | 12.36 (10.28-14.85) | **7.68** | 15.95 (13.24-19.22) |
| **65 years and older** |  |  |  |  |  |  |  |  |
| Primary care | 429.47 | 424.39 (386.68-465.79) | **378.70** | 441.47 (401.54-485.38) | **321.80** | 375.33 (339.52-414.91) | **341.18** | 403.44 (363.96-447.21) |
| Specialty care | 236.63 | 231.86 (209.31-256.84) | **224.53** | 276.64 (249.72-306.47) | 235.53 | 246.72 (222.70-273.33) | 262.78 | 247.48 (222.11-275.81) |
| Virtual visit | 6.22 | 4.87 (2.43-7.30) | **400.81** | 5.51 (3.08-7.94) | **379.59** | 5.05 (2.62-7.48) | **387.93** | 5.17 (2.67-7.66) |
| ED visits | 121.08 | 124.44 (107.85-143.58) | **64.91** | 126.77 (105.99-151.71) | **58.84** | 98.68 (81.11-120.05) | **55.13** | 114.53 (93.50-140.34) |
| Hospitalizations | 71.70 | 73.91 (64.11-85.21) | **38.58** | 72.41 (60.63-86.53) | **34.52** | 54.75 (45.03-66.56) | **32.81** | 65.23 (53.27-79.90) |
| ***Sex*** | | | | | | | | |
| **Female** |  |  |  |  |  |  |  |  |
| Primary care | 139.20 | 132.54 (119.21-147.35) | 124.97 | 138.87 (124.41-155.01) | **103.22** | 116.63 (104.01-130.78) | **108.99** | 130.58 (115.37-147.84) |
| Specialty care | 71.05 | 68.68 (63.13-74.73) | **67.37** | 81.45 (74.74-88.76) | 68.99 | 72.70 (66.24-79.80) | 79.71 | 74.75 (67.63-82.63) |
| Virtual visit | 2.51 | 2.69 (2.26-3.12) | **129.92** | 2.90 (2.45-3.36) | **119.20** | 2.94 (2.40-3.48) | **123.52** | 3.13 (2.46-3.81) |
| ED visits | 39.13 | 42.83 (35.85-51.18) | **21.26** | 39.55 (33.10-47.26) | **17.63** | 30.74 (25.72-36.74) | **17.30** | 38.67 (32.25-46.36) |
| Hospitalizations | 20.34 | 22.15 (18.46-26.57) | **10.37** | 19.63 (16.36-23.55) | **9.19** | 14.76 (12.31-17.71) | **9.07** | 19.13 (15.89-23.01) |
| **Male** |  |  |  |  |  |  |  |  |
| Primary care | 148.60 | 144.35 (130.53-159.64) | **127.90** | 147.21 (132.79-163.21) | **108.36** | 126.13 (113.45-140.24) | **116.26** | 138.65 (123.92-155.16) |
| Specialty care | 86.66 | 82.52 (74.36-91.57) | **77.91** | 96.91 (87.16-107.75) | 81.40 | 86.24 (77.23-96.30) | 92.15 | 87.72 (77.70-99.05) |
| Virtual visit | 2.93 | 2.85 (2.40-3.31) | **136.76** | 2.99 (2.51-3.46) | **130.36** | 3.01 (2.48-3.55) | **135.11** | 3.25 (2.60-3.89) |
| ED visits | 40.50 | 42.15 (36.96-48.07) | **22.63** | 40.30 (34.85-46.60) | **20.51** | 32.26 (27.70-37.57) | **18.37** | 38.93 (33.35-45.46) |
| Hospitalizations | 20.64 | 20.47 (17.79-23.55) | **12.19** | 19.96 (16.99-23.46) | **10.39** | 15.46 (13.01-18.38) | **9.79** | 18.59 (15.52-22.28) |

*In bold: observed rates outside the projected 95% confidence intervals of projected rates were considered as significantly different.

**Table S4**. Observed and projected monthly rates and 95% confidence intervals (CI) in subgroups estimated by ARIMA Models for outpatient and inpatient (emergency department visits and hospitalizations) care visits for **asthma**: rates were calculated as the number of events per 100,000 people at risk. Similar periods in previous years (2016-2019) were used to calculate projected rates.

| Population  Rates | Observed | Projected  (95% CI) | Observed | Projected  (95% CI) | Observed | Projected  (95% CI) | Observed | Projected  (95% CI) |
| --- | --- | --- | --- | --- | --- | --- | --- | --- |
|  | **Pre-COVID**  Jan-Feb 2020 | | **Wave I**  Mar-May 2020 | | **Summer Lull**  Jun-Aug 2020 | | **Wave II** Sep 2020 - Mar 2021 | |
| ***Age***, years | | | | | | | | |
| **18-24 years old** |  |  |  |  |  |  |  |  |
| Primary care | 181.13 | 182.38 (163.43-203.53) | **229.09** | 180.06 (160.74-201.70) | 174.26 | 182.30 (162.08-205.05) | 167.65 | 181.28 (161.16-203.92) |
| Specialty care | 50.60 | 46.88 (41.77-51.99) | 50.26 | 51.91 (46.69-57.13) | 56.27 | 52.83 (46.98-58.67) | **58.49** | 47.99 (41.60-54.38) |
| Virtual visit | 4.36 | 5.05 (3.78-6.32) | **204.73** | 5.10 (3.76-6.44) | **168.46** | 5.79 (4.24-7.34) | **160.00** | 6.54 (4.62-8.46) |
| ED visits | 20.19 | 21.29 (17.67-25.65) | **14.04** | 22.93 (18.90-27.81) | **11.43** | 21.09 (17.33-25.66) | **11.79** | 24.03 (19.70-29.30) |
| Hospitalizations | 1.01 | 1.07 (0.36-1.77) | **0.48** | 1.42 (0.69-2.15) | 0.30 | 0.89 (0.11-1.66) | **0.52** | 1.34 (0.55-2.14) |
| **25-34 years old** |  |  |  |  |  |  |  |  |
| Primary care | 181.96 | 165.32 (147.32-185.52) | **241.72** | 170.50 (151.24-192.22) | 168.74 | 153.09 (135.41-173.08) | 171.05 | 167.60 (147.29-190.77) |
| Specialty care | 50.53 | 47.28 (42.02-53.19) | 49.20 | 48.99 (43.40-55.32) | 52.85 | 46.84 (41.37-53.03) | **63.07** | 47.99 (42.19-54.60) |
| Virtual visit | 5.71 | 5.78 (4.97-6.58) | **212.28** | 5.91 (4.97-6.84) | **166.33** | 6.21 (5.03-7.38) | **170.57** | 6.70 (5.14-8.26) |
| ED visits | 17.02 | 16.62 (14.03-19.68) | **13.73** | 17.62 (14.84-20.92) | **9.15** | 15.27 (12.86-18.13) | **9.95** | 18.63 (15.66-22.15) |
| Hospitalizations | 0.99 | 1.00 (0.63-1.59) | **0.48** | 1.09 (0.68-1.75) | **0.51** | 0.88 (0.55-1.42) | **0.59** | 1.17 (0.72-1.88) |
| **35-49 years old** |  |  |  |  |  |  |  |  |
| Primary care | 203.40 | 193.54 (173.55-215.84) | **286.24** | 184.69 (164.07-207.91) | **189.39** | 159.75 (140.86-181.18) | 184.39 | 181.92 (160.10-206.71) |
| Specialty care | 78.88 | 74.32 (66.79-82.71) | 77.31 | 76.92 (68.65-86.18) | **82.75** | 70.70 (62.92-79.44) | **92.83** | 74.81 (66.21-84.54) |
| Virtual visit | 4.87 | 5.19 (4.35-6.03) | **268.29** | 5.31 (4.41-6.21) | **204.92** | 5.67 (4.60-6.74) | **201.46** | 6.36 (5.02-7.71) |
| ED visits | 15.42 | 15.31 (12.19-19.24) | 11.90 | 15.10 (11.83-19.28) | **8.26** | 13.71 (10.65-17.64) | **8.14** | 15.47 (11.94-20.05) |
| Hospitalizations | 1.04 | 1.20 (0.74-1.66) | 0.80 | 1.22 (0.76-1.68) | **0.61** | 1.22 (0.74-1.69) | **0.57** | 1.19 (0.72-1.67) |
| **50-64 years old** |  |  |  |  |  |  |  |  |
| Primary care | 214.88 | 202.58 (177.59-231.09) | **286.07** | 204.35 (177.96-234.66) | 201.99 | 175.62 (152.29-202.53) | 194.60 | 194.62 (167.48-226.23) |
| Specialty care | 120.90 | 114.94 (106.21-124.39) | 117.92 | 127.99 (117.54-139.36) | 126.21 | 115.59 (105.86-126.21) | **139.88** | 121.44 (110.75-133.19) |
| Virtual visit | 3.89 | 4.06 (3.32-4.80) | **297.85** | 4.08 (3.32-4.84) | **244.88** | 4.28 (3.45-5.10) | **234.73** | 4.60 (3.67-5.53) |
| ED visits | 13.37 | 15.38 (12.11-19.54) | **9.29** | 13.23 (10.28-17.03) | **6.23** | 10.62 (8.19-13.78) | **5.76** | 12.29 (9.42-16.03) |
| Hospitalizations | 1.41 | 1.34 (0.92-1.94) | **0.85** | 1.28 (0.88-1.86) | **0.59** | 0.87 (0.60-1.25) | **0.62** | 1.11 (0.76-1.61) |
| **65 years and older** |  |  |  |  |  |  |  |  |
| Primary care | 211.73 | 201.80 (177.37-229.60) | 234.27 | 226.99 (193.04-267.04) | 187.36 | 187.24 (156.91-223.42) | 191.89 | 205.68 (171.30-247.03) |
| Specialty care | 173.70 | 172.15 (157.13-188.60) | **172.83** | 211.98 (193.32-232.45) | 183.10 | 188.03 (171.31-206.39) | 202.10 | 191.49 (173.53-211.34) |
| Virtual visit | 2.88 | 2.65 (1.90-3.40) | **301.80** | 3.90 (3.12-4.68) | **279.28** | 4.13 (3.16-5.10) | **277.00** | 3.84 (2.59-5.09) |
| ED visits | 13.52 | 14.71 (12.15-17.82) | **7.01** | 13.89 (11.31-17.06) | **4.88** | 10.47 (8.44-12.99) | **5.31** | 13.09 (10.52-16.28) |
| Hospitalizations | 2.60 | 3.18 (2.27-4.45) | **1.62** | 3.02 (2.15-4.24) | **1.06** | 2.03 (1.44-2.87) | **1.08** | 2.59 (1.81-3.71) |
| ***Sex*** | | | | | | | | |
| **Female** |  |  |  |  |  |  |  |  |
| Primary care | 231.35 | 214.42 (188.51-243.88) | **308.64** | 222.56 (192.38-257.51) | 213.29 | 189.62 (162.57-221.17) | 207.44 | 214.85 (182.96-252.39) |
| Specialty care | 126.32 | 122.15 (113.14-131.88) | 126.09 | 136.69 (125.59-148.77) | 135.26 | 125.54 (114.86-137.22) | **149.54** | 128.70 (117.29-141.22) |
| Virtual visit | 4.54 | 4.48 (3.72-5.24) | **326.16** | 4.85 (3.80-5.91) | **264.95** | 5.04 (3.34-6.74) | **257.04** | 5.30 (2.88-7.72) |
| ED visits | 19.16 | 18.84 (15.53-22.86) | **13.71** | 20.11 (16.42-24.62) | **8.59** | 15.77 (12.82-19.41) | **8.98** | 19.04 (15.30-23.71) |
| Hospitalizations | 2.06 | 2.28 (1.79-2.91) | **1.25** | 2.44 (1.92-3.12) | **0.91** | 1.70 (1.33-2.17) | **0.90** | 2.16 (1.69-2.77) |
| **Male** |  |  |  |  |  |  |  |  |
| Primary care | 171.72 | 169.58 (156.41-183.85) | **211.76** | 165.51 (151.98-180.25) | 159.67 | 148.11 (135.49-161.90) | 160.96 | 160.20 (146.47-175.21) |
| Specialty care | 77.88 | 78.57 (72.58-85.06) | **74.82** | 83.89 (76.72-91.72) | 79.73 | 76.21 (69.41-83.68) | **90.27** | 79.84 (72.43-88.02) |
| Virtual visit | 4.00 | 4.49 (3.95-5.04) | **205.22** | 4.32 (3.73-4.91) | **175.38** | 4.64 (3.92-5.37) | **175.75** | 5.03 (4.10-5.96) |
| ED visits | 11.15 | 12.01 (10.14-14.22) | **7.54** | 11.13 (9.39-13.18) | **6.31** | 9.73 (8.21-11.52) | **6.13** | 11.40 (9.61-13.51) |
| Hospitalizations | 0.85 | 0.94 (0.67-1.32) | **0.54** | 0.90 (0.64-1.26) | **0.39** | 0.74 (0.53-1.04) | **0.49** | 0.90 (0.64-1.26) |

*In bold: observed rates outside the projected 95% confidence intervals of projected rates were considered as significantly different.

**Table S5**. Observed and projected monthly rates and 95% confidence intervals (CI) in subgroups estimated by ARIMA Models for outpatient and inpatient (emergency department visits and hospitalizations) care visits for **angina**: rates were calculated as the number of events per 100,000 people at risk. Similar periods in previous years (2016-2019) were used to calculate projected rates.

| Population  Rates | Observed | Projected  (95% CI) | Observed | Projected  (95% CI) | Observed | Projected  (95% CI) | Observed | Projected  (95% CI) |
| --- | --- | --- | --- | --- | --- | --- | --- | --- |
|  | **Pre-COVID**  Jan-Feb 2020 | | **Wave I**  Mar-May 2020 | | **Summer Lull**  Jun-Aug 2020 | | **Wave II** Sep 2020 - Mar 2021 | |
| ***Age***, years | | | | | | | | |
| **25-34 years old** |  |  |  |  |  |  |  |  |
| Primary care | 1.49 | 1.40 (0.95-2.05) | 1.21 | 1.40 (0.95-2.05) | 1.27 | 1.40 (0.95-2.05) | 1.17 | 1.40 (0.95-2.05) |
| Specialty care | 3.31 | 2.79 (2.09-3.73) | 2.69 | 3.12 (2.32-4.18) | 3.03 | 2.68 (2.00-3.59) | 3.28 | 2.92 (2.17-3.93) |
| Virtual visit | 0.00 | 0.02 (-0.06-0.10) | **2.28** | 0.02 (-0.06-0.10) | **2.66** | 0.02 (-0.06-0.10) | **2.58** | 0.02 (-0.06-0.10) |
| ED visits | 0.24 | 0.14 (-0.10-0.39) | 0.12 | 0.17 (-0.08-0.42) | 0.31 | 0.14 (-0.10-0.39) | 0.12 | 0.16 (-0.08-0.41) |
| Hospitalizations | 0.05 | 0.09 (-0.04-0.21) | 0.08 | 0.09 (-0.03-0.22) | 0.06 | 0.09 (-0.04-0.21) | 0.06 | 0.08 (-0.04-0.21) |
| **35-49 years old** |  |  |  |  |  |  |  |  |
| Primary care | 8.78 | 8.40 (7.18-9.82) | **6.96** | 8.39 (7.16-9.82) | 7.65 | 7.53 (6.41-8.86) | 7.84 | 7.88 (6.66-9.32) |
| Specialty care | 15.67 | 14.77 (12.37-17.64) | **12.92** | 16.06 (13.41-19.25) | 13.96 | 15.42 (12.79-18.57) | 15.56 | 15.89 (13.14-19.21) |
| Virtual visit | 0.12 | 0.10 (-0.01-0.21) | **11.67** | 0.06 (-0.06-0.18) | **13.52** | 0.05 (-0.07-0.18) | **14.11** | 0.05 (-0.07-0.18) |
| ED visits | 1.99 | 2.53 (1.89-3.38) | **1.42** | 2.53 (1.89-3.38) | 1.94 | 2.53 (1.89-3.38) | 1.97 | 2.53 (1.89-3.38) |
| Hospitalizations | 0.65 | 0.75 (0.51-1.10) | **0.48** | 0.76 (0.52-1.13) | **0.51** | 0.78 (0.52-1.15) | 0.59 | 0.78 (0.52-1.15) |
| **50-64 years old** |  |  |  |  |  |  |  |  |
| Primary care | 36.40 | 36.56 (33.34-40.09) | **34.54** | 39.25 (35.68-43.18) | 36.15 | 35.37 (31.57-39.63) | 38.04 | 35.41 (30.78-40.74) |
| Specialty care | 68.56 | 65.79 (56.97-74.61) | **58.14** | 71.92 (63.06-80.78) | **60.98** | 71.55 (62.60-80.49) | 67.16 | 71.78 (62.53-81.03) |
| Virtual visit | 0.59 | 0.51 (-0.10-1.11) | **53.85** | 0.34 (-0.30-0.99) | **61.81** | 0.29 (-0.39-0.96) | **63.60** | 0.36 (-0.32-1.04) |
| ED visits | 9.45 | 9.37 (8.45-10.38) | **7.92** | 9.45 (8.49-10.51) | 8.15 | 9.07 (8.12-10.13) | **7.97** | 9.12 (8.15-10.20) |
| Hospitalizations | 3.21 | 3.26 (2.72-3.90) | **2.06** | 3.09 (2.57-3.72) | 2.78 | 2.99 (2.44-3.67) | 2.48 | 2.89 (2.27-3.68) |
| **65 years and older** |  |  |  |  |  |  |  |  |
| Primary care | 106.10 | 102.88 (95.07-111.32) | **99.70** | 113.91 (103.80-125.03) | 101.48 | 102.83 (92.01-114.91) | 96.77 | 101.21 (90.02-113.80) |
| Specialty care | 156.88 | 150.62 (132.89-168.34) | **141.81** | 184.63 (166.74-202.52) | 165.11 | 184.97 (161.27-208.68) | 171.30 | 178.03 (153.49-202.58) |
| Virtual visit | 1.24 | 0.46 (0.06-3.33) | **138.50** | 0.46 (0.06-3.33) | **163.19** | 0.46 (0.06-3.33) | **157.87** | 0.46 (0.06-3.33) |
| ED visits | 21.73 | 21.69 (19.66-23.92) | **17.15** | 22.83 (20.66-25.22) | 20.16 | 21.04 (18.97-23.35) | **18.72** | 20.96 (18.77-23.42) |
| Hospitalizations | 6.99 | 7.61 (6.45-8.98) | **5.47** | 7.73 (6.52-9.17) | 7.33 | 6.53 (5.33-8.01) | 6.49 | 6.21 (4.71-8.19) |
| ***Sex*** | | | | | | | | |
| **Female** |  |  |  |  |  |  |  |  |
| Primary care | 23.04 | 21.89 (19.79-24.21) | **21.62** | 24.17 (21.81-26.77) | 23.16 | 21.53 (19.41-23.89) | 22.56 | 21.76 (19.49-24.29) |
| Specialty care | 38.18 | 35.17 (28.67-41.67) | **33.48** | 41.93 (35.40-48.47) | 38.15 | 42.40 (35.85-48.95) | 40.64 | 41.88 (34.95-48.81) |
| Virtual visit | 0.42 | 0.19 (-0.34-0.73) | **32.26** | 0.19 (-0.34-0.73) | **38.17** | 0.19 (-0.34-0.73) | **38.43** | 0.19 (-0.34-0.73) |
| ED visits | 5.69 | 5.80 (5.13-6.56) | **4.36** | 5.81 (5.11-6.61) | 5.25 | 5.40 (4.67-6.24) | 5.11 | 5.44 (4.60-6.43) |
| Hospitalizations | 1.87 | 2.32 (1.82-2.95) | **1.34** | 2.32 (1.82-2.95) | **1.73** | 2.32 (1.82-2.95) | **1.70** | 2.32 (1.82-2.95) |
| **Male** |  |  |  |  |  |  |  |  |
| Primary care | 47.68 | 45.62 (42.44-49.04) | **44.28** | 51.17 (47.29-55.37) | 44.89 | 45.24 (41.09-49.81) | 44.75 | 45.07 (40.89-49.68) |
| Specialty care | 75.88 | 71.36 (62.86-79.85) | **66.84** | 84.37 (75.78-92.95) | 74.82 | 83.86 (73.62-94.09) | 79.77 | 82.40 (71.73-93.07) |
| Virtual visit | 0.49 | 0.38 (0.09-1.61) | **63.56** | 0.18 (0.04-0.95) | **74.00** | 0.21 (0.03-1.27) | **73.14** | 0.21 (0.03-1.27) |
| ED visits | 9.86 | 9.55 (8.52-10.71) | **8.07** | 10.29 (9.17-11.56) | 8.96 | 9.75 (8.65-10.99) | **8.37** | 9.69 (8.50-11.05) |
| Hospitalizations | 3.21 | 3.44 (2.89-4.10) | **2.41** | 3.44 (2.88-4.10) | 3.25 | 3.35 (2.80-4.02) | 2.78 | 3.22 (2.68-3.86) |

*In bold: observed rates outside the projected 95% confidence intervals of projected rates were considered as significantly different.

**Table S6**. Observed and projected monthly rates and 95% confidence intervals (CI) in subgroups estimated by ARIMA Models for outpatient and inpatient (emergency department visits and hospitalizations) care visits for **congestive heart failure** **(CHF)**: rates were calculated as the number of events per 100,000 people at risk. Similar periods in previous years (2016-2019) were used to calculate projected rates.

| Population  Rates | Observed | Projected  (95% CI) | Observed | Projected  (95% CI) | Observed | Projected  (95% CI) | Observed | Projected  (95% CI) |
| --- | --- | --- | --- | --- | --- | --- | --- | --- |
|  | **Pre-COVID**  Jan-Feb 2020 | | **Wave I**  Mar-May 2020 | | **Summer Lull**  Jun-Aug 2020 | | **Wave II** Sep 2020 - Mar 2021 | |
| ***Age***, years | | | | | | | | |
| **18-24 years old** |  |  |  |  |  |  |  |  |
| Primary care | 1.05 | 0.72 (0.32-1.60) | 0.70 | 0.65 (0.28-1.48) | 0.89 | 0.83 (0.36-1.92) | 0.74 | 0.74 (0.31-1.75) |
| Specialty care | 3.59 | 3.26 (2.38-4.46) | 2.93 | 3.68 (2.68-5.04) | 3.57 | 3.40 (2.48-4.65) | 3.60 | 3.42 (2.50-4.68) |
| Virtual visit | 0.04 | 0.02 (-0.02-0.07) | **2.37** | 0.02 (-0.03-0.06) | **2.57** | 0.01 (-0.04-0.05) | **2.51** | 0.01 (-0.03-0.06) |
| ED visits | 0.16 | 0.20 (-0.04-0.44) | 0.13 | 0.20 (-0.04-0.44) | 0.14 | 0.20 (-0.04-0.44) | 0.15 | 0.20 (-0.04-0.44) |
| Hospitalizations | 0.08 | 0.14 (-0.12-0.39) | 0.13 | 0.13 (-0.13-0.39) | 0.16 | 0.13 (-0.13-0.39) | 0.08 | 0.13 (-0.14-0.40) |
| **25-34 years old** |  |  |  |  |  |  |  |  |
| Primary care | 1.44 | 1.67 (1.01-2.75) | 1.71 | 1.67 (1.01-2.75) | 1.65 | 1.67 (1.01-2.75) | 1.76 | 1.67 (1.01-2.75) |
| Specialty care | 6.61 | 6.09 (4.97-7.45) | 5.47 | 6.15 (5.01-7.56) | 6.43 | 5.76 (4.69-7.08) | 6.77 | 6.18 (5.02-7.61) |
| Virtual visit | 0.00 | 0.04 (-0.04-0.12) | **4.36** | 0.02 (-0.06-0.10) | **4.80** | 0.03 (-0.06-0.11) | **4.70** | 0.04 (-0.04-0.12) |
| ED visits | 0.43 | 0.56 (0.22-0.91) | 0.28 | 0.58 (0.22-0.94) | 0.51 | 0.54 (0.17-0.91) | 0.48 | 0.54 (0.17-0.91) |
| Hospitalizations | 0.38 | 0.47 (0.19-0.75) | 0.25 | 0.38 (0.09-0.67) | 0.45 | 0.40 (0.09-0.70) | 0.37 | 0.40 (0.09-0.71) |
| **35-49 years old** |  |  |  |  |  |  |  |  |
| Primary care | 6.13 | 6.32 (5.38-7.41) | 6.42 | 6.39 (5.44-7.50) | **7.63** | 6.15 (5.24-7.22) | **7.62** | 6.20 (5.28-7.28) |
| Specialty care | 19.91 | 18.94 (16.74-21.15) | **16.81** | 19.53 (17.19-21.87) | 20.02 | 17.85 (15.34-20.37) | 20.47 | 18.75 (16.11-21.39) |
| Virtual visit | 0.12 | 0.12 (0.03-0.21) | **14.84** | 0.08 (-0.01-0.18) | **16.18** | 0.07 (-0.03-0.16) | **16.03** | 0.08 (-0.02-0.17) |
| ED visits | 2.25 | 2.12 (1.57-2.66) | 1.91 | 2.01 (1.43-2.58) | 2.09 | 1.98 (1.38-2.57) | 1.90 | 1.98 (1.38-2.57) |
| Hospitalizations | 1.62 | 1.61 (1.08-2.13) | 1.39 | 1.53 (0.96-2.10) | 1.75 | 1.48 (0.89-2.08) | 1.55 | 1.48 (0.88-2.08) |
| **50-64 years old** |  |  |  |  |  |  |  |  |
| Primary care | 33.43 | 32.10 (29.06-35.13) | 34.60 | 35.43 (32.37-38.48) | **37.05** | 33.08 (29.96-36.20) | 35.83 | 32.91 (29.64-36.19) |
| Specialty care | 69.89 | 66.13 (59.38-73.66) | **65.13** | 74.77 (67.14-83.28) | 70.55 | 67.58 (60.68-75.27) | 75.64 | 70.31 (62.92-78.58) |
| Virtual visit | 0.46 | 0.34 (0.07-0.61) | **59.95** | 0.34 (0.06-0.61) | **63.33** | 0.33 (0.05-0.61) | **62.68** | 0.32 (0.03-0.61) |
| ED visits | 10.93 | 11.53 (10.18-12.88) | **8.59** | 11.56 (10.19-12.94) | 9.90 | 10.17 (8.71-11.63) | **8.70** | 10.89 (9.27-12.50) |
| Hospitalizations | 8.07 | 7.89 (7.07-8.81) | **6.90** | 7.87 (7.02-8.83) | 7.55 | 7.23 (6.42-8.15) | **6.66** | 7.78 (6.90-8.76) |
| **65 years and older** |  |  |  |  |  |  |  |  |
| Primary care | 389.45 | 382.35 (357.11-409.36) | 408.17 | 427.04 (397.07-459.29) | 421.72 | 397.98 (367.19-431.35) | 420.30 | 388.34 (357.00-422.47) |
| Specialty care | 329.34 | 316.21 (291.25-343.30) | **313.37** | 376.91 (346.30-410.22) | 351.86 | 342.70 (312.25-376.12) | 361.01 | 351.14 (313.97-392.83) |
| Virtual visit | 2.42 | 1.63 (0.20-3.06) | **415.30** | 1.63 (0.20-3.06) | **414.31** | 1.63 (0.20-3.06) | **391.56** | 1.63 (0.20-3.06) |
| ED visits | 96.26 | 96.44 (89.31-104.15) | **65.45** | 104.78 (96.66-113.58) | **76.64** | 88.25 (80.57-96.66) | **75.72** | 93.08 (83.60-103.64) |
| Hospitalizations | 77.63 | 77.93 (72.96-83.23) | **54.96** | 83.19 (77.82-88.92) | **62.39** | 70.69 (65.98-75.73) | **62.74** | 77.08 (71.69-82.88) |
| ***Sex*** | | | | | | | | |
| **Female** |  |  |  |  |  |  |  |  |
| Primary care | 93.75 | 92.96 (86.29-100.15) | 98.60 | 100.61 (93.38-108.40) | **102.85** | 95.02 (88.18-102.38) | **103.37** | 95.01 (87.82-102.81) |
| Specialty care | 77.93 | 76.32 (70.92-82.13) | **72.82** | 86.92 (80.15-94.26) | 82.91 | 80.78 (73.99-88.20) | 86.13 | 82.65 (75.55-90.42) |
| Virtual visit | 0.63 | 0.74 (0.50-0.98) | **99.38** | 0.71 (0.47-0.95) | **101.48** | 0.74 (0.50-0.98) | **97.41** | 0.82 (0.58-1.07) |
| ED visits | 24.06 | 24.06 (22.06-26.06) | **15.36** | 26.03 (23.97-28.10) | **18.32** | 22.65 (20.40-24.91) | **18.48** | 24.26 (21.72-26.80) |
| Hospitalizations | 19.58 | 19.47 (17.86-21.22) | **13.53** | 20.56 (18.86-22.42) | **15.35** | 17.78 (16.30-19.38) | **15.65** | 19.51 (17.88-21.29) |
| **Male** |  |  |  |  |  |  |  |  |
| Primary care | 97.40 | 94.10 (87.35-101.36) | 101.81 | 106.85 (98.49-115.94) | 106.19 | 98.29 (89.40-108.06) | **106.19** | 95.94 (86.93-105.89) |
| Specialty care | 115.72 | 109.48 (100.55-119.21) | **109.45** | 132.46 (121.63-144.25) | 121.72 | 117.76 (107.14-129.43) | 126.96 | 121.64 (109.78-134.82) |
| Virtual visit | .73 | 0.78 (0.55-1.02) | **123.07** | 0.76 (0.52-0.99) | **123.84** | 0.66 (0.42-0.90) | **119.13** | 0.82 (0.58-1.06) |
| ED visits | 24.93 | 25.32 (23.56-27.22) | **18.85** | 26.68 (24.71-28.80) | 21.78 | 22.66 (20.79-24.69) | **20.78** | 23.62 (21.26-26.25) |
| Hospitalizations | 19.43 | 19.39 (18.05-20.83) | **14.90** | 21.17 (19.67-22.78) | 17.05 | 17.97 (16.59-19.47) | **16.55** | 19.75 (18.17-21.47) |

*In bold: observed rates outside the projected 95% confidence intervals of projected rates were considered as significantly different.

**Table S7**. Observed and projected monthly rates and 95% confidence intervals (CI) in subgroups estimated by ARIMA Models for outpatient and inpatient (emergency department visits and hospitalizations) care visits for **diabetes**: rates were calculated as the number of events per 100,000 people at risk. Similar periods in previous years (2016-2019) were used to calculate projected rates.

| Population  Rates | Observed | Projected  (95% CI) | Observed | Projected  (95% CI) | Observed | Projected  (95% CI) | Observed | Projected  (95% CI) |
| --- | --- | --- | --- | --- | --- | --- | --- | --- |
|  | **Pre-COVID**  Jan-Feb 2020 | | **Wave I**  Mar-May 2020 | | **Summer Lull**  Jun-Aug 2020 | | **Wave II** Sep 2020 - Mar 2021 | |
| ***Age***, years | | | | | | | | |
| **18-24 years old** |  |  |  |  |  |  |  |  |
| Primary care | 60.13 | 62.06 (54.33-70.89) | **56.16** | 64.70 (56.64-73.91) | 63.51 | 62.97 (55.13-71.93) | **71.23** | 60.95 (53.18-69.85) |
| Specialty care | 96.16 | 92.11 (84.23-99.98) | 95.81 | 94.78 (86.76-102.80) | **105.68** | 94.69 (86.39-102.99) | **106.22** | 95.78 (86.64-104.91) |
| Virtual visit | **2.87** | 2.01 (1.23-2.79) | **109.46** | 2.22 (1.44-3.01) | **128.88** | 2.31 (1.51-3.12) | **131.14** | 2.46 (1.61-3.30) |
| ED visits | 10.22 | 10.06 (8.39-12.07) | **7.10** | 10.26 (8.55-12.31) | 8.83 | 10.18 (8.49-12.22) | **7.93** | 10.23 (8.51-12.28) |
| Hospitalizations | 5.73 | 5.50 (4.38-6.91) | **4.49** | 5.75 (4.51-7.32) | 5.16 | 5.61 (4.36-7.21) | 4.82 | 5.60 (4.35-7.20) |
| **25-34 years old** |  |  |  |  |  |  |  |  |
| Primary care | 160.41 | 150.33 (137.25-164.65) | **147.02** | 162.10 (147.75-177.84) | 166.93 | 156.04 (141.83-171.67) | **185.19** | 157.13 (142.23-173.61) |
| Specialty care | 214.79 | 213.83 (199.88-228.76) | 226.65 | 224.20 (209.15-240.34) | **246.03** | 226.88 (209.70-245.48) | **250.40** | 214.59 (195.49-235.66) |
| Virtual visit | 3.71 | 3.25 (2.46-4.04) | **259.93** | 3.28 (2.48-4.09) | **315.51** | 3.19 (2.39-3.99) | **323.19** | 3.57 (2.76-4.38) |
| ED visits | 8.69 | 8.47 (7.26-9.88) | **6.27** | 8.47 (7.25-9.90) | 7.75 | 8.46 (7.24-9.89) | 7.46 | 8.46 (7.24-9.89) |
| Hospitalizations | 3.94 | 4.05 (3.24-5.06) | **3.14** | 4.05 (3.24-5.06) | 4.20 | 4.05 (3.24-5.06) | 3.87 | 4.05 (3.24-5.06) |
| **35-49 years old** |  |  |  |  |  |  |  |  |
| Primary care | 730.93 | 682.13 (632.32-735.86) | **619.90** | 736.55 (681.53-796.02) | 704.66 | 697.02 (642.50-756.17) | 758.65 | 716.21 (656.74-781.11) |
| Specialty care | 298.90 | 288.54 (266.54-312.37) | 294.51 | 309.13 (284.49-335.91) | **317.21** | 290.06 (265.58-316.80) | **330.67** | 298.03 (270.73-328.10) |
| Virtual visit | 8.56 | 8.20 (6.98-9.42) | **604.06** | 8.54 (7.32-9.77) | **714.43** | 8.69 (7.41-9.98) | **729.90** | 9.27 (7.54-11.00) |
| ED visits | 10.92 | 10.08 (8.68-11.71) | **8.67** | 10.44 (8.95-12.17) | 10.82 | 10.37 (8.82-12.18) | 9.25 | 10.33 (8.79-12.15) |
| Hospitalizations | 4.07 | 3.96 (3.15-4.77) | 3.73 | 3.84 (3.02-4.65) | 4.27 | 3.96 (3.13-4.78) | 3.72 | 3.98 (3.13-4.82) |
| **50-64 years old** |  |  |  |  |  |  |  |  |
| Primary care | **2,237.10** | 2,035.06 (1,884.32-2,197.85) | **1,848.36** | 2,258.07 (2,089.21-2,440.58) | 2,116.10 | 2,131.22 (1,971.07-2,304.38) | 2,261.85 | 2,208.29 (2,033.22-2,398.73) |
| Specialty care | 629.50 | 616.46 (577.83-657.68) | **562.96** | 665.13 (618.27-715.56) | 623.40 | 610.75 (563.57-661.89) | 659.06 | 635.74 (582.44-693.98) |
| Virtual visit | 20.88 | 18.54 (14.35-23.96) | **1,522.48** | 21.47 (16.24-28.40) | **1,783.31** | 23.82 (16.69-34.03) | **1,806.93** | 28.39 (16.93-47.82) |
| ED visits | 14.94 | 14.21 (12.94-15.60) | **10.42** | 14.77 (13.43-16.23) | 13.47 | 14.26 (12.88-15.78) | 12.90 | 14.20 (12.77-15.78) |
| Hospitalizations | 5.54 | 4.88 (3.99-5.96) | 4.22 | 4.34 (3.51-5.37) | 4.87 | 4.40 (3.53-5.47) | 4.62 | 4.42 (3.55-5.50) |
| **65 years and older** |  |  |  |  |  |  |  |  |
| Primary care | **3,746.98** | 3,484.66 (3,244.52-3,742.58) | **3,085.42** | 4,075.21 (3,792.31-4,379.22) | 3,600.36 | 3,832.92 (3,565.92-4,119.92) | 3,841.06 | 3,904.46 (3,617.66-4,214.44) |
| Specialty care | 951.04 | 899.83 (841.39-962.32) | **846.48** | 1,078.79 (996.21-1,168.28) | 982.12 | 980.70 (896.42-1,072.91) | 1,030.46 | 1,010.30 (915.46-1,115.08) |
| Virtual visit | 23.88 | 20.63 (16.13-25.13) | **2,423.16** | 21.70 (17.15-26.25) | **2,815.75** | 22.66 (18.10-27.21) | **2,795.24** | 24.74 (20.14-29.34) |
| ED visits | 21.83 | 22.24 (19.97-24.78) | **14.38** | 22.72 (20.35-25.37) | **18.15** | 22.34 (19.87-25.12) | **17.21** | 22.09 (19.53-24.98) |
| Hospitalizations | 7.63 | 7.23 (6.23-8.40) | **6.16** | 7.41 (6.38-8.61) | 6.90 | 7.15 (6.16-8.31) | 6.93 | 7.49 (6.43-8.73) |
| ***Sex*** | | | | | | | | |
| **Female** |  |  |  |  |  |  |  |  |
| Primary care | **1381.46** | 1,252.02 (1,155.69-1,356.38) | **1,156.77** | 1,462.77 (1,349.87-1,585.12) | 1,368.86 | 1,385.41 (1,278.32-1,501.48) | 1,463.17 | 1,413.85 (1,298.47-1,539.68) |
| Specialty care | 467.90 | 448.39 (419.64-479.11) | **448.26** | 516.73 (479.06-557.38) | 504.84 | 479.97 (442.10-521.07) | 525.37 | 489.87 (448.43-535.19) |
| Virtual visit | 13.20 | 11.64 (8.99-15.07) | **1,031.40** | 13.38 (10.18-17.59) | **1,238.97** | 14.47 (10.55-19.86) | **1,248.09** | 16.87 (11.44-24.89) |
| ED visits | 12.92 | 12.23 (11.35-13.18) | **8.24** | 12.63 (11.72-13.60) | **10.94** | 12.16 (11.29-13.10) | **10.12** | 12.26 (11.38-13.21) |
| Hospitalizations | 4.79 | 4.71 (4.20-5.30) | **3.78** | 4.70 (4.18-5.28) | 4.69 | 4.71 (4.18-5.29) | 4.39 | 4.81 (4.27-5.42) |
| **Male** |  |  |  |  |  |  |  |  |
| Primary care | **1,851.44** | 1,701.61 (1,584.88-1,826.94) | **1,523.07** | 1,898.00 (1,765.42-2,040.53) | 1,728.96 | 1,784.60 (1,658.79-1,919.95) | 1,864.18 | 1,848.58 (1,711.40-1,996.95) |
| Specialty care | 516.13 | 504.24 (476.11-534.04) | **456.79** | 546.16 (510.04-584.87) | 512.00 | 502.00 (464.25-542.82) | 542.74 | 523.73 (481.35-569.88) |
| Virtual visit | 14.16 | 13.17 (11.89-14.46) | **1,229.26** | 13.75 (12.45-15.05) | **1,408.95** | 14.91 (12.99-16.82) | **1,423.41** | 16.21 (13.18-19.24) |
| ED visits | 14.88 | 14.81 (13.85-15.84) | **11.41** | 15.24 (14.21-16.34) | **13.86** | 14.99 (13.90-16.18) | **12.96** | 14.86 (13.76-16.05) |
| Hospitalizations | 5.99 | 5.80 (5.19-6.41) | **4.96** | 5.68 (5.06-6.29) | 5.48 | 5.34 (4.73-5.96) | 5.22 | 5.77 (5.15-6.39) |

*In bold: observed rates outside the projected 95% confidence intervals of projected rates were considered as significantly different.

**Table S8**. Observed and projected monthly rates and 95% confidence intervals (CI) in subgroups estimated by ARIMA Models for outpatient and inpatient (emergency department visits and hospitalizations) care visits for **hypertension**: rates were calculated as the number of events per 100,000 people at risk. Similar periods in previous years (2016-2019) were used to calculate projected rates.

| Population  Rates | Observed | Projected  (95% CI) | Observed | Projected  (95% CI) | Observed | Projected  (95% CI) | Observed | Projected  (95% CI) |
| --- | --- | --- | --- | --- | --- | --- | --- | --- |
|  | **Pre-COVID**  Jan-Feb 2020 | | **Wave I**  Mar-May 2020 | | **Summer Lull**  Jun-Aug 2020 | | **Wave II** Sep 2020 - Mar 2021 | |
| ***Age***, years | | | | | | | | |
| **18-24 years old** |  |  |  |  |  |  |  |  |
| Primary care | 56.78 | 54.57 (48.85-60.96) | **38.44** | 57.62 (51.52-64.45) | **44.63** | 55.23 (49.38-61.77) | 52.72 | 51.94 (46.38-58.16) |
| Specialty care | 12.48 | 10.66 (8.15-13.95) | 9.04 | 11.65 (8.90-15.24) | 8.29 | 9.92 (7.58-12.98) | 11.26 | 10.45 (7.95-13.72) |
| Virtual visit | 0.48 | 0.27 (-0.21-0.76) | **26.29** | 0.27 (-0.21-0.76) | **27.79** | 0.27 (-0.21-0.76) | **30.80** | 0.27 (-0.21-0.76) |
| ED visits | 1.86 | 1.32 (0.83-2.09) | 0.81 | 1.24 (0.78-1.99) | 1.30 | 1.23 (0.77-1.98) | 1.32 | 1.23 (0.77-1.98) |
| Hospitalizations | 0.08 | 0.09 (-0.06-0.24) | 0.03 | 0.10 (-0.05-0.26) | 0.22 | 0.10 (-0.06-0.26) | 0.10 | 0.10 (-0.06-0.27) |
| **25-34 years old** |  |  |  |  |  |  |  |  |
| Primary care | 188.94 | 186.17 (174.46-198.67) | **143.96** | 203.27 (190.28-217.15) | **158.80** | 177.81 (164.81-191.83) | 178.81 | 188.62 (173.57-204.99) |
| Specialty care | 30.39 | 27.02 (22.44-32.54) | 25.45 | 29.46 (24.46-35.48) | 27.77 | 26.45 (21.96-31.85) | **33.79** | 27.28 (22.53-33.03) |
| Virtual visit | 2.48 | 2.26 (1.66-2.87) | **99.34** | 2.40 (1.73-3.07) | **108.20** | 2.62 (1.78-3.45) | **116.20** | 2.84 (1.77-3.91) |
| ED visits | 4.42 | 4.44 (3.45-5.43) | **2.76** | 4.47 (3.44-5.49) | 3.77 | 3.87 (2.75-4.99) | 4.62 | 4.59 (3.33-5.85) |
| Hospitalizations | 0.31 | 0.28 (0.02-0.54) | 0.25 | 0.33 (0.06-0.59) | 0.29 | 0.36 (0.10-0.63) | 0.32 | 0.29 (0.02-0.56) |
| **35-49 years old** |  |  |  |  |  |  |  |  |
| Primary care | 891.21 | 860.77 (800.84-925.18) | **733.45** | 907.34 (841.18-978.72) | 738.11 | 784.47 (713.53-862.46) | 814.12 | 847.01 (767.53-934.80) |
| Specialty care | 71.01 | 66.83 (58.72-76.06) | **58.23** | 75.41 (66.24-85.85) | 61.96 | 64.19 (56.38-73.09) | 75.45 | 67.90 (59.14-77.99) |
| Virtual visit | 8.65 | 7.95 (7.02-8.88) | **510.44** | 8.77 (7.78-9.76) | **530.70** | 9.21 (8.04-10.38) | **553.05** | 10.11 (8.65-11.57) |
| ED visits | 15.74 | 15.44 (13.94-16.95) | **10.27** | 15.45 (13.91-16.99) | **11.36** | 13.38 (11.75-15.02) | **13.39** | 15.67 (13.88-17.46) |
| Hospitalizations | 0.98 | 0.82 (0.49-1.36) | 0.56 | 0.93 (0.56-1.54) | 0.95 | 0.76 (0.46-1.27) | 0.92 | 0.88 (0.53-1.47) |
| **50-64 years old** |  |  |  |  |  |  |  |  |
| Primary care | 2,278.94 | 2,158.81 (1,992.83-2,338.61) | **1,955.98** | 2,330.68 (2,151.49-2,524.79) | 1,990.17 | 2,106.56 (1,944.60-2,282.01) | 2,139.44 | 2,197.42 (2,022.61-2,387.46) |
| Specialty care | 174.06 | 168.61 (155.53-182.78) | **129.48** | 181.77 (167.21-197.60) | **141.08** | 162.78 (148.90-177.95) | 171.41 | 179.30 (159.51-201.63) |
| Virtual visit | 22.39 | 20.45 (17.99-22.92) | **1,352.56** | 22.43 (19.49-25.36) | **1,442.09** | 21.22 (17.11-25.34) | **1,448.04** | 24.28 (20.05-28.51) |
| ED visits | 30.16 | 28.68 (25.59-32.14) | **20.03** | 29.15 (25.97-32.73) | 21.44 | 22.87 (20.37-25.67) | 26.03 | 28.93 (25.74-32.52) |
| Hospitalizations | 1.68 | 1.51 (1.00-2.03) | 1.32 | 1.53 (1.01-2.06) | 1.40 | 1.52 (0.99-2.05) | 1.52 | 1.51 (0.98-2.05) |
| **65 years and older** |  |  |  |  |  |  |  |  |
| Primary care | 3,880.08 | 3,728.35 (3,425.66-4,057.78) | **3,535.24** | 4,279.53 (3,929.70-4,660.51) | 3,672.67 | 3,849.74 (3,532.33-4,195.67) | 3,937.28 | 4,001.83 (3,653.29-4,384.23) |
| Specialty care | 369.70 | 350.01 (317.09-386.35) | **284.08** | 418.65 (378.28-463.34) | 335.81 | 373.59 (333.60-418.39) | 384.20 | 376.62 (327.93-432.73) |
| Virtual visit | **27.82** | 24.86 (22.02-27.70) | **2,552.86** | 29.76 (26.42-33.10) | **2,701.81** | 31.01 (26.16-35.86) | **2,650.51** | 34.38 (27.50-41.25) |
| ED visits | 63.64 | 61.85 (55.65-68.05) | **39.34** | 63.34 (56.94-69.73) | **45.54** | 52.48 (46.04-58.92) | 55.88 | 61.87 (55.40-68.34) |
| Hospitalizations | 4.23 | 4.28 (3.32-5.52) | **2.83** | 4.41 (3.41-5.72) | 3.67 | 3.92 (3.03-5.08) | 4.32 | 4.21 (3.24-5.46) |
| ***Sex*** | | | | | | | | |
| **Female** |  |  |  |  |  |  |  |  |
| Primary care | 1,680.00 | 1,611.76 (1,486.40-1,747.69) | **1,504.67** | 1,843.75 (1,700.27-1,999.34) | 1,565.55 | 1,637.82 (1,485.89-1,805.29) | 1,683.79 | 1,700.68 (1,536.10-1,883.11) |
| Specialty care | 147.83 | 142.68 (131.26-155.09) | **113.46** | 164.52 (150.71-179.60) | **131.36** | 148.97 (135.60-163.65) | 153.66 | 155.76 (138.25-175.56) |
| Virtual visit | 12.57 | 11.69 (10.31-13.06) | **1078.97** | 13.77 (12.27-15.27) | **1151.25** | 14.93 (13.06-16.79) | **1,151.62** | 17.30 (14.78-19.81) |
| ED visits | 30.51 | 29.66 (27.01-32.30) | **19.13** | 31.06 (28.37-33.74) | **22.90** | 26.29 (23.39-29.19) | **27.44** | 30.86 (27.57-34.15) |
| Hospitalizations | 1.86 | 1.83 (1.37-2.46) | **1.25** | 1.99 (1.48-2.67) | 1.69 | 1.74 (1.28-2.36) | 1.87 | 1.95 (1.41-2.70) |
| **Male** |  |  |  |  |  |  |  |  |
| Primary care | 1,711.63 | 1,628.07 (1,502.51-1,764.13) | **1,470.36** | 1,781.12 (1,643.17-1,930.66) | 1,500.41 | 1,586.31 (1,463.17-1,719.82) | 1635.52 | 1,697.38 (1,558.03-1,849.46) |
| Specialty care | 151.94 | 146.75 (133.79-160.97) | **117.18** | 164.75 (149.79-181.21) | **130.85** | 146.81 (132.00-163.29) | 156.22 | 156.16 (136.68-178.52) |
| Virtual visit | **16.49** | 14.52 (13.00-16.05) | **1026.17** | 17.94 (16.20-19.69) | **1,082.25** | 19.21 (16.39-22.04) | **1,086.61** | 21.21 (16.95-25.47) |
| ED visits | 22.40 | 21.33 (19.11-23.54) | **14.51** | 21.37 (19.16-23.58) | **15.19** | 17.47 (15.25-19.68) | **19.05** | 21.34 (19.12-23.56) |
| Hospitalizations | 1.45 | 1.42 (1.09-1.84) | **1.04** | 1.44 (1.11-1.88) | 1.25 | 1.29 (0.99-1.68) | 1.41 | 1.37 (1.05-1.80) |

*In bold: observed rates outside the projected 95% confidence intervals of projected rates were considered as significantly different.

**Table S9**. Observed and projected monthly rates and 95% confidence intervals (CI) in subgroups estimated by ARIMA Models for outpatient and inpatient (emergency department visits and hospitalizations) care visits for **epilepsy**: rates were calculated as the number of events per 100,000 people at risk. Similar periods in previous years (2016-2019) were used to calculate projected rates.

| Population  Rates | Observed | Projected  (95% CI) | Observed | Projected  (95% CI) | Observed | Projected  (95% CI) | Observed | Projected  (95% CI) |
| --- | --- | --- | --- | --- | --- | --- | --- | --- |
|  | **Pre-COVID**  Jan-Feb 2020 | | **Wave I**  Mar-May 2020 | | **Summer Lull**  Jun-Aug 2020 | | **Wave II** Sep 2020 - Mar 2021 | |
| ***Age***, years | | | | | | | | |
| **18-24 years old** |  |  |  |  |  |  |  |  |
| Primary care | 25.12 | 26.29 (22.72-30.44) | **23.45** | 27.27 (23.49-31.64) | 29.37 | 25.69 (21.90-30.13) | **30.19** | 25.20 (21.39-29.69) |
| Specialty care | 72.77 | 70.56 (62.93-79.12) | 76.52 | 72.49 (64.32-81.70) | 77.61 | 72.35 (63.89-81.94) | **85.60** | 69.39 (61.06-78.87) |
| Virtual visit | 2.87 | 2.25 (1.36-3.15) | **76.85** | 2.60 (1.62-3.58) | **86.30** | 2.19 (0.96-3.41) | **93.25** | 2.32 (0.91-3.74) |
| ED visits | 11.63 | 11.56 (9.64-13.86) | **8.77** | 12.40 (10.33-14.90) | **9.40** | 12.87 (10.72-15.46) | **9.93** | 12.26 (10.19-14.75) |
| Hospitalizations | 3.43 | 3.09 (2.03-4.16) | 2.42 | 3.13 (2.06-4.21) | 2.46 | 3.13 (2.04-4.23) | 2.84 | 3.13 (2.00-4.26) |
| **25-34 years old** |  |  |  |  |  |  |  |  |
| Primary care | 24.72 | 25.21 (22.57-28.15) | 23.59 | 24.75 (22.06-27.77) | **28.46** | 23.88 (20.71-27.54) | **29.28** | 24.24 (20.20-29.09) |
| Specialty care | 58.08 | 53.08 (47.06-59.86) | 56.94 | 57.34 (50.84-64.67) | 60.78 | 55.49 (49.19-62.59) | **66.86** | 55.97 (49.42-63.39) |
| Virtual visit | 2.43 | 2.50 (1.72-3.27) | **58.61** | 2.76 (1.98-3.54) | **70.81** | 2.82 (2.04-3.60) | **75.06** | 2.94 (2.15-3.73) |
| ED visits | 8.59 | 9.01 (7.63-10.63) | **6.67** | 9.00 (7.61-10.64) | 7.84 | 9.01 (7.59-10.70) | **7.54** | 9.04 (7.61-10.73) |
| Hospitalizations | 3.02 | 2.57 (1.89-3.51) | **1.70** | 2.57 (1.89-3.51) | 2.81 | 2.57 (1.89-3.51) | 2.28 | 2.57 (1.89-3.51) |
| **35-49 years old** |  |  |  |  |  |  |  |  |
| Primary care | 23.14 | 22.26 (19.57-25.31) | 23.00 | 22.73 (19.96-25.88) | **25.45** | 21.89 (19.22-24.92) | **26.59** | 21.49 (18.85-24.51) |
| Specialty care | 42.37 | 41.74 (37.46-46.49) | 42.02 | 44.75 (40.13-49.91) | 43.20 | 41.08 (36.72-45.94) | **48.52** | 42.58 (37.89-47.86) |
| Virtual visit | 1.61 | 1.57 (1.06-2.08) | **47.01** | 1.64 (1.11-2.17) | **53.32** | 1.71 (1.13-2.29) | **57.38** | 1.83 (1.16-2.49) |
| ED visits | 6.26 | 5.83 (5.00-6.80) | **5.15** | 6.76 (5.80-7.88) | **5.81** | 6.79 (5.82-7.91) | 5.90 | 6.43 (5.50-7.50) |
| Hospitalizations | 2.10 | 1.91 (1.41-2.58) | 1.47 | 1.91 (1.41-2.58) | 1.78 | 1.91 (1.41-2.58) | 1.86 | 1.91 (1.41-2.58) |
| **50-64 years old** |  |  |  |  |  |  |  |  |
| Primary care | 26.82 | 27.21 (24.63-30.07) | 27.43 | 26.43 (23.86-29.26) | **30.30** | 25.85 (23.19-28.82) | **30.73** | 24.86 (21.98-28.14) |
| Specialty care | 41.88 | 40.19 (36.71-44.00) | 41.14 | 45.20 (41.11-49.70) | 42.25 | 39.76 (36.07-43.83) | **47.50** | 41.66 (37.60-46.17) |
| Virtual visit | 1.10 | 1.05 (0.77-1.33) | **46.71** | 1.05 (0.75-1.35) | **53.45** | 1.23 (0.88-1.57) | **55.58** | 1.23 (0.82-1.65) |
| ED visits | 5.61 | 5.16 (4.45-5.98) | **4.45** | 5.31 (4.57-6.17) | 5.15 | 5.53 (4.76-6.42) | 4.86 | 5.07 (4.36-5.90) |
| Hospitalizations | 2.32 | 2.53 (2.03-3.15) | **1.98** | 2.53 (2.03-3.15) | 2.33 | 2.53 (2.03-3.15) | 2.25 | 2.53 (2.03-3.15) |
| **65 years and older** |  |  |  |  |  |  |  |  |
| Primary care | 26.68 | 25.69 (23.51-28.07) | 29.37 | 27.28 (24.96-29.82) | **32.88** | 26.47 (24.19-28.97) | **31.80** | 25.91 (23.63-28.39) |
| Specialty care | 40.24 | 38.83 (35.10-42.95) | **41.99** | 46.70 (42.22-51.66) | 45.38 | 42.01 (37.97-46.47) | **48.92** | 42.59 (38.41-47.23) |
| Virtual visit | .71 | 0.69 (0.44-0.93) | **48.29** | 0.73 (0.46-1.01) | **55.43** | 0.79 (0.47-1.11) | **56.33** | 0.85 (0.53-1.17) |
| ED visits | 4.63 | 4.63 (3.88-5.52) | 3.87 | 4.54 (3.80-5.43) | 4.52 | 4.61 (3.82-5.57) | 4.45 | 4.57 (3.73-5.61) |
| Hospitalizations | 2.88 | 3.37 (2.70-4.22) | **2.48** | 3.46 (2.77-4.33) | 2.97 | 3.45 (2.75-4.32) | 3.26 | 3.43 (2.73-4.29) |
| ***Sex*** | | | | | | | | |
| **Female** |  |  |  |  |  |  |  |  |
| Primary care | 22.62 | 22.25 (20.86-23.72) | 23.71 | 23.37 (21.76-25.10) | **27.37** | 23.19 (21.43-25.09) | **27.01** | 22.28 (20.57-24.14) |
| Specialty care | 45.88 | 44.27 (40.43-48.47) | 47.16 | 49.20 (44.71-54.15) | 48.48 | 45.10 (40.65-50.03) | **53.25** | 45.81 (41.19-50.94) |
| Virtual visit | 1.51 | 1.40 (1.10-1.69) | **50.77** | 1.52 (1.21-1.83) | **58.26** | 1.63 (1.27-1.99) | **60.88** | 1.73 (1.31-2.15) |
| ED visits | 5.84 | 5.44 (4.83-6.05) | **3.96** | 5.71 (5.10-6.32) | **4.80** | 5.89 (5.27-6.50) | **4.69** | 5.48 (4.86-6.10) |
| Hospitalizations | 2.14 | 2.24 (1.83-2.74) | **1.61** | 2.24 (1.83-2.74) | 1.96 | 2.24 (1.83-2.74) | 2.05 | 2.24 (1.83-2.74) |
| **Male** |  |  |  |  |  |  |  |  |
| Primary care | 28.18 | 26.81 (24.93-28.84) | 27.74 | 28.75 (26.68-30.98) | **31.21** | 27.07 (24.87-29.45) | **32.37** | 26.54 (24.27-29.01) |
| Specialty care | 49.39 | 50.58 (46.63-54.88) | 48.64 | 52.26 (47.63-57.35) | 51.53 | 48.73 (44.18-53.74) | **57.31** | 49.22 (44.44-54.51) |
| Virtual visit | 1.59 | 1.57 (1.21-1.93) | **53.81** | 1.67 (1.31-2.04) | **62.20** | 1.74 (1.37-2.10) | **65.89** | 1.81 (1.44-2.18) |
| ED visits | 7.57 | 7.64 (6.77-8.62) | **6.74** | 8.27 (7.33-9.33) | **7.40** | 8.41 (7.45-9.49) | 7.36 | 8.04 (7.12-9.07) |
| Hospitalizations | 3.13 | 3.05 (2.60-3.58) | **2.31** | 3.05 (2.59-3.58) | 2.93 | 3.04 (2.58-3.58) | 2.85 | 3.03 (2.58-3.57) |

*In bold: observed rates outside the projected 95% confidence intervals of projected rates were considered as significantly different.


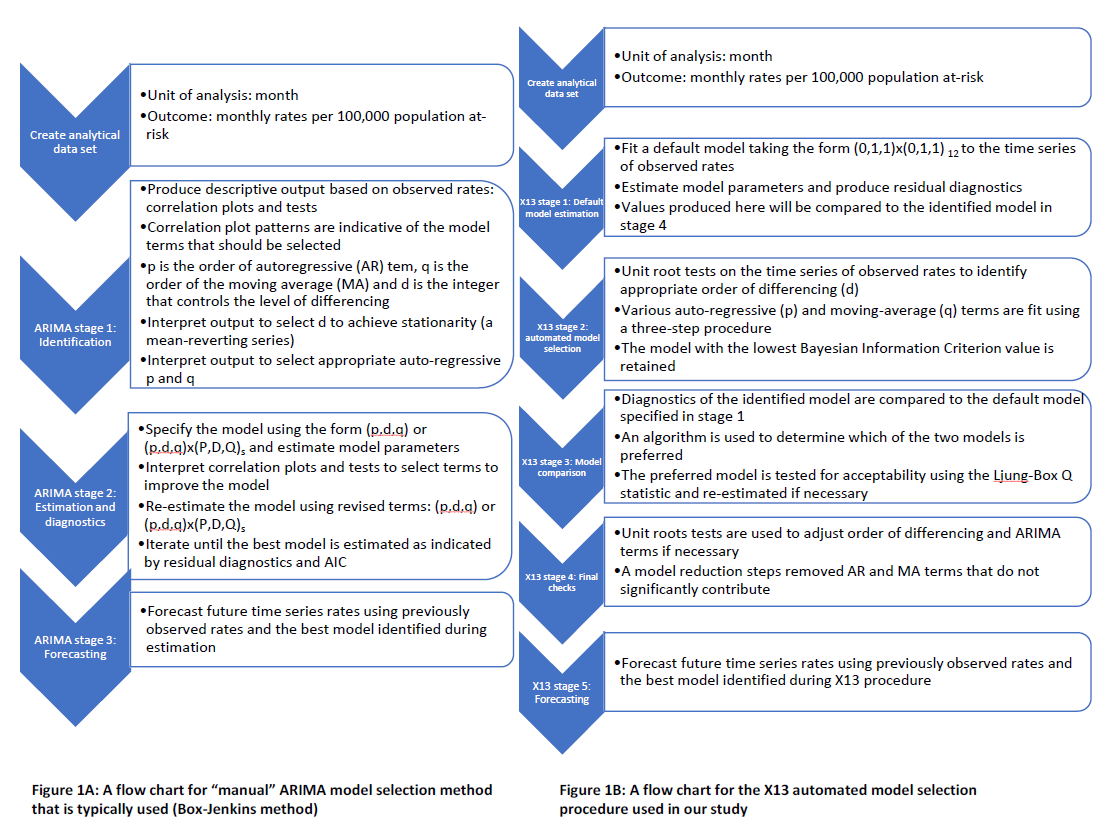


**Figure S1**: (A) A flow chart for “manual” ARIMA model selection method that is typically used (Box-Jenkins method); (B) A flow chart for the X13 automated model selection procedure used in our study.

**References**:

1. Box GEP, Jenkins GM, Reinsel GC: **Time series analysis: forecasting and control**: John Wiley; 2008.

2. **Time series research staff, Center for Statistical Research and Methodology, U.S. Census Bureau. Reference Manual for X-13ARIMA-SEATS** In*.* Washington, DC 20233 2017.

3. Dagum EB: **The X11ARIMA/88 Seasonal Adjustment Method - Foundations and User's Manual. Time Series Research and Analysis Division.** . In: *Statistics Canada Technical Report.* 1988.

4. Gomez V, Maravall A: **Automatic modeling methods for univariate series**. In: *A Course in Time Series Analysis.* edn. Edited by Pena D, Tiao GC, Tsay RS. New York, NY: J. Wiley and Sons; 2001.

5. Huang YT, Lee YC, Hsiao CJ: **Hospitalization for ambulatory-care-sensitive conditions in Taiwan following the SARS outbreak: a population-based interrupted time series study**. *J Formos Med Assoc* 2009, **108**(5):386-394.

6. **Improving health care data in Ontario.** *ICES investigative report Toronto: Institute for Clinical Evaluative Sciences* 2005.

7. Schull MJ, Azimaee M, Marra M, Cartagena RG, Vermeulen MJ, Ho M, Guttmann A: **ICES: Data, Discovery, Better Health**. *Int J Popul Data Sci* 2020, **4**(2):1135.

8. Juurlink D PC, Croxford R, Chong A, Austin P, Tu J, Laupacis A. : **Canadian Institute for Health Information Discharge Abstract Database: A Validation Study** In*.* Toronto: Institute for Clinical Evaluative Sciences; 2006: 69.

9. Goel V, Canadian Medical Association., Institute for Clinical Evaluative Sciences in Ontario.: **Patterns of health care in Ontario**, 2nd edn. Ottawa: Published by the Canadian Medical Association [for] the Institute for Clinical Evaluative Sciences in Ontario; 1996.

10. Quan H, Smith M, Bartlett-Esquilant G, Johansen H, Tu K, Lix L, Hypertension O, Surveillance T: **Mining administrative health databases to advance medical science: geographical considerations and untapped potential in Canada**. *The Canadian journal of cardiology* 2012, **28**(2):152-154.

11. Schneeweiss S, Avorn J: **A review of uses of health care utilization databases for epidemiologic research on therapeutics**. *Journal of clinical epidemiology* 2005, **58**(4):323-337.

12. Doyle CM, Lix LM, Hemmelgarn BR, Paterson JM, Renoux C: **Data variability across Canadian administrative health databases: Differences in content, coding, and completeness**. *Pharmacoepidemiology and drug safety* 2020, **29 Suppl 1**:68-77.

13. **COVID19 Integrated Testing Data (C19INTGR)** [<https://www.hdrn.ca/en/inventory/4761/>]

14. **Ontario Laboratories Information System COVID-19 data (OLISC19)** [<https://www.hdrn.ca/en/inventory/4506/>]

15. **Case and Contact Management System (CCM)** [<https://www.hdrn.ca/en/inventory/4763/>]

16. **Text Table A. International Statistical Classification of Diseases and Related Health Problems, 10th Revision, Canada (ICD-10-CA) codes used to define ambulatory care sensitive conditions** [<https://www150.statcan.gc.ca/n1/pub/82-003-x/2017012/article/54891/tbl/ttbla-eng.htm>]
